# An interactive key to Central European species of the *Pteromalus albipennis* species group and other species of the genus (Hymenoptera: Chalcidoidea: Pteromalidae), with the description of a new species

**DOI:** 10.3897/BDJ.6.e27722

**Published:** 2018-11-14

**Authors:** Fabian Klimmek, Hannes Baur

**Affiliations:** 1 Department of Invertebrates, Natural History Museum Bern, Bern, Switzerland Department of Invertebrates, Natural History Museum Bern Bern Switzerland; 2 Institute of Ecology and Evolution, University of Bern, Bern, Switzerland Institute of Ecology and Evolution, University of Bern Bern Switzerland

**Keywords:** Diptera, microtomy, paraspiracular inclination, Pteromalinae, *P. achillei*, *P. caudiger*, *P. cingulipes*, *P. eudecipiens*, *P. intermedius*, *P. patro*, *P. puparum*, *P. temporalis*, shape principal component analysis, taxonomy, Tephritidae

## Abstract

**Background:**

Parasitoid wasps of the genus *Pteromalus* play an important role in biological pest control, however, the genus includes a large number of cryptic species, which makes reliable identification difficult. The latest identification key dates back to Graham (1969) and since then many new species have been described and nomenclatural changes proposed.

**New information:**

Here we present an interactive and fully illustrated identification key in Xper3 for 27 species of the *Pteromalus albipennis* species group as well as for 18 similar species. In addition to qualitative traits, a large set of body measurements is incorporated in the key. We also explored a new set of qualitative features on the propodeum and metasternum. During field work, a new species of the *P. albipennis* species group, *P. capito* Baur sp. n., could be reared from flower heads of Asteraceae, which is described here. It looks very similar to *P. albipennis* and *P. cingulipes*, however, several qualitative characters and body ratios distinguish it clearly from the most similar species.

## Introduction

Recently developed software for the creation of biological identification keys like Xper3 (Vignes-Lebbe et al. 2017), Lucid (Lucidcentral.org 2017) or Delta (Dallwitz et al. 2018) could have the potential to replace traditional paper-based keys. They allow the integration of large amounts of quantitative data and high quality photographs as well as accurate drawings and precise descriptions. Since these keys are able to offer a multi-access way to identification, even damaged or juvenile specimens can be identified by avoiding inapplicable characters. Traditional dichotomous keys in these cases often lead to a dead end. Additionally, these keys can be easily edited and continued by other users, thus providing the possibility for constant improvement. The digital nature of such keys furthermore allows quick and cheap publication via the internet and easy access for interested users worldwide. Examples are given on the XPER depository website (Key to Nature 2010) and the Lucid Key Server (Lucidcentral.org 2017). While the XPER depository website is outdated and provides only two keys, the Lucid Key Server comes with over 100 different keys covering many different groups of organisms. Even a very limited key to three genera of Pteromalidae in Southeast Asia (Fisher et al. 2005) and to a genus of Braconidae in Costa Rica (Marsh and Wild 2013) are available. Major disadvantages of Lucid are the relatively high cost. On the other hand, Xper3 works independently in every browser and is available at no charge. However, it is not open source and is licensed under the Creative Commons Attribution-Noncommercial-ShareAlike 3.0 license France. It was developed by the Laboratory of Informatics and Systematics of University Pierre et Marie Curie (Paris 6).

Here we created an interactive identification key in Xper3 for European *Pteromalus* with special focus on the *P. albipennis* species group. The latest key for the identification of *Pteromalus* is a monograph on the Pteromalidae of North-Western Europe (Graham 1969). It includes over 800 species in 196 genera with accurately illustrated keys to species level. However, the key is outdated by now and no recent alternatives are available. With our interactive key, we want to create a basic descriptive dataset on which other taxonomists and interested laymen can build. Despite the range of species that is covered by Graham’s work and more recent discoveries (Gijswijt 1999, Graham and Gijswijt 1991, Janzon 1984, Gijswijt 1972), there are still new species to be found even in relatively well studied areas like Central Europe (Baur 2015). In this context, a new species from Switzerland and Northern Italy is described here and also included in the key. Nowadays, advanced computer technology, measurement procedures and equipment allow more sophisticated ways to include quantitative characters, which greatly enhance the delimitation and recognition of cryptic species. So far, mostly body ratios and easily accessible morphological traits have been studied and used to delimit species of Pteromalidae. The examination of more hidden traits, which require partial or total dissection of the specimens, have not been used in previous keys, although these may greatly benefit species identification. Similarly, maceration has never been applied to study internal or hidden structures for species delimitation. In this case, maceration refers to a treatment where specimens are soaked in 10% KOH for several hours, rendering the body parts transparent and permitting better examination of internal structures and preparation of slide material (Noyes 1982).

Parasitoid wasps of the genus *Pteromalus* belong to the family Pteromalidae (Insecta: Hymenoptera: Chalcidoidea). The genus contains 371 known species in Europe (Noyes 2016) and is the most speciose of this family. Many of these belong to cryptic species complexes with hardly any morphologically distinguishing features (Graham 1969). All species of the genus *Pteromalus* parasitise larvae and pupae of different insect hosts, including Diptera, Lepidoptera and Coleoptera (Noyes 2016, Janzon 1984). To simplify and recapitulate the huge diversity of this group, Graham (1969) established a framework of species groups. The *Pteromalus albipennis* group consists of about two dozen species which share the following characters: the lower edge of the antennal toruli is placed distinctly above the level of the ventral edge of the eyes, the anterior margin of the clypeus is moderately incised, the costal setal line of the fore wing is often interrupted medially or before the middle and the posterior part of the plica of the propodeum converges strongly towards the median line (Graham 1969). Members of the *P. albipennis* species group mainly parasitise fruit flies of the family Tephritidae (Diptera) (e.g. Janzon 1984). They oviposit into flower heads of Asteraceae (e.g. *Achillea* sp., *Centaurea* sp., *Cirsium* sp.). The adult wasps lay their eggs on to the larvae by penetrating the wall of the flower head with their ovipositor.

## Materials and methods

### Creation of the key

The key was created in Xper3 version 1.4.0. Pictures could be uploaded as JPEGs and are hosted on a free Dropbox account. The original TIF-files are deposited on zenodo.org. Descriptors were ranked with the built-in ranking system of Xper3 (rank 1 = little important, 5 = highly important) according to their distinct nature and relative simplicity to study. Measurements, which were taken for all species, were read in as quantitative data with fixed ranges. Completeness of the database was checked using the inbuilt tools for analysis of the data.

### Use of Xper3

As the official user manual of Xper3 (Vignes-Lebbe et al. 2017) is somewhat rudimentary, we hence include here a short account of the main features and possibilities offered by Xper3 and how we used them.

The user of Xper3 has several tabs to edit the descriptive data in the software. In the “Items” section all taxa are listed and can be provided with additional detailed information (e.g. distribution, first author, habitat, indicator values etc.) and with illustrating material like pictures, movies, sound files or any other file (e.g. maps, text files). In addition, new taxa can be added and edited in the same way as already existing taxa. The “Descriptive model” section lists all descriptors and can also be edited as described for the “Items” section. States for each descriptor can be edited or new ones can be added.

Xper3 has the possibility to weight the descriptors. This is a great way to simplify and speed up the identification process, allowing quick and reliable results. For instance, problematic (e.g. shape of propodeal foramen) or rather difficult (e.g. degree of paraspiracular inclination) characters can be weighted less, which reduces the chances for users to deal with them already at the beginning of the identification process. However, there is no guarantee that such characters will never appear in the top part of the list.

The section “Description” offers the possibility to assign each descriptor the respective states for each item.

Xper3 comes with some diagnostic tools to check the completeness of the database or to analyse the data. A view of the complete description matrix with the possibility to search for undescribed items and the revision of these is a great help to make sure that each taxon is completely described.

Furthermore, it is possible to select various items or assign them to groups for comparison. The output is a matrix, which also shows all descriptor states of every included item. In addition, the states for each descriptor, where the items differ or are congruent, are displayed separately.

The tool “Checkbase” is an automated search for various errors in the database, for instance items with identical descriptions, undescribed or inapplicable descriptors and descriptors described as unknown. A direct link to these errors provides a handy means for quick and easy revision. Finally, it is possible to generate different kinds of keys. Besides the interactive version, flat or tree-shaped dichotomous versions can be generated in various output formats (HTML, PDF, SDD, Zip archive etc.).

To work with the key, one selects any character which seems conspicuous or unique to the specimen and decides which state is appropriate. Of course, it is recommended to start with the top characters, as they have been weighted higher by the author for good reason. Once a selection is made, the software will exclude every taxon which does not fit this set of states and give a list of the remaining possible names. If more than one name remains, more characters have to be studied. If only one name remains, this should normally be the name one is looking for. This can be checked by comparing the specimen with all the pictures linked to this taxon and the complete diagnosis. The section “History” provides a helpful means to look up the identification process and correct potential mismatches without resetting the complete selection.

### Validity of the key

The taxa to be included in the key were based on a list of valid European species for the genus *Pteromalus* from the Universal Chalcidoidea Database (Noyes 2016). A first list yielded 374 species names, from which we excluded 301 names *a priori*. These names did not belong to species of our target species group, are of dubious status or belong to species of which the type specimens are lost.

Of the remaining 73 names, we included 28 species belonging to the *P. albipennis* species group (sensu Graham 1969). *Pteromalus annae*, *P. arnicae* and *P. sonchi* were excluded, since we regard these as possible synonyms of *P. caudiger*, *P. albipennis* and *P. intermedius*. *Pteromalus leucanthemi* might be considered a synonym of *P. albipennis* (Lutz 2014). *P. cingulipes*, currently a synonym of *P. albipennis*, has been demonstrated to be a valid species (Maletti et al. in press) and is therefore treated separately. *P. ametrus* is morphologically extremely close to *P. temporalis* but since our paper is not a revision of the taxon, we consider it as a valid species.

Of the remaining 47 names, we included 9 species from 8 species groups (sensu Graham 1969) as representatives. Additionally, 9 names that either belong to species regarded as *species sola* (Graham 1969) or to recently described species were selected. Finally, we included one new species, *Pteromalus capito* Baur sp. n. In total, 47 species were included (Table 1).

If the key leads to species names representing whole species groups (e.g. *altus*, *sequester* etc.), the resulting identification has to be treated with caution. In this case, we refer to (Graham 1969) for a sound identification.

Nomenclature and classification of Pteromalidae follow Noyes (2016). Terminol­ogy of body parts follows Gibson et al. (1997) and for terms concerning sculpture of the integu­ment and for some particular expressions used in the description, we refer to Graham (1969). The separation of the plica of the propodeum into an anterior and a poste­rior part is according to Graham (1969). The above mentioned progress in chalcid taxonomy applies merely to females. Due to their short life cycle, males can exhibit much more variation in important traits without suffering too many restrictions in fitness and are therefore much harder to identify reliably (Graham 1969, Bouček and Rasplus 1991). In addition, males are not available for many species. Therefore, only females were examined in this work. For the identification of males, we refer to Graham (1969).

### Material examined

All specimens relevant to this work are listed in appendix A. Examined specimens are deposited in the following collections (abbreviations according to Noyes (2016)): Biological Museum (Entomology), Lund University, Lund, Sweden (LUZM); Natural History Mu­seum Bern, Bern, Switzerland (NMBE); Oxford University Museum of Natural History, University of Oxford, Oxford, UK (OUM), Swedish Museum of Natural History, Stockholm, Sweden (NHRS).

### Preparation

**Figure 1.**
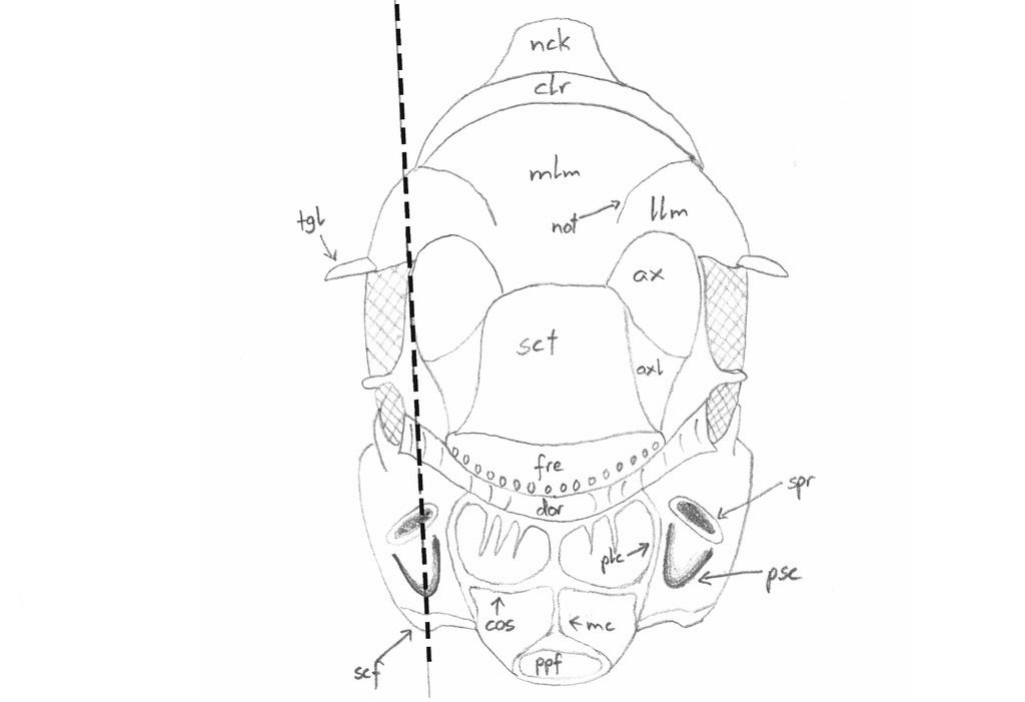
Pteromalinae sp., schematic dorsal view of the mesosoma. The line indicates the orientation of grinding. After Gibson et al. (1997).

Most characters were studied on dried and card mounted specimens (Noyes 1982). Concerning character selection and definitions of character states, we often followed Graham (1969). The descriptive data of *P. aartseni* were based on the original description by Gijswijt (1972). For better examination of the back of the head, slides were prepared using the method described by Noyes (1982). Microtomy was applied to access the paraspiracular inclination at optimal conditions for digital microphotography. This is a new character, which describes the shape of the paraspiracular sulcus. It is best seen in lateral or dorsolateral view. However, hind legs, wings or the propodeal callus may mask the view, which makes it very difficult to spot the differences. Processed specimens are listed in Suppl. material 5. Specimens were all preserved in 90% ethanol and stored in a refrigerator before processing. LR White (soft grade) (London Resin Company Ltd., Reading, Berkshire), a resin commonly used in immunocytochemistry (e.g. Wynford-Thomas et al. 1986), served as the tissue embedding medium. The specimens were first immersed in 100% ethanol for one hour to completely dehydrate. Then head, legs, wings and gaster were removed and preserved again in 90% ethanol. The remaining body was transferred to LR White for three hours. The LR White bath was exchanged three times. After the third replacement, 10 ml LR White and one drop of LR White accelerator were mixed thoroughly and the specimen was placed within the resin until it became viscous and the object kept its position. The medium was allowed to polymerise hermetically sealed in a water bath overnight. Polymerised resin blocks were grinded using a Leica RM 2145 microtome. The specimen was orientated in a way that the glass blade grinded it laterally. The material was grinded until the spiracle and the paraspiracular sulcus were truncated (Fig. 1). Resin blocks were afterwards immersed in 100% ethanol overnight to dissolve from the body. Then all parts of the respective specimen were dried using the AXA-method (Alcohol/Xylene-Amyl acetate method), a procedure which minimises shrinking and deformation (Achterberg van 2009). Dried body parts were glued on a card point with fish glue (Syndetikon) (Noyes 1982) and stored in the collection of the NMBE. The drying and mounting procedure was applied to all ethanol specimens which are accounted for in this work.

### Measurements

Characters were selected and measured according to the procedure described in Baur (2015) (Table 2). The distances were measured using ImageJ, version 1.51j8 (Schneider et al. 2012). To avoid variation due to fluctuating asymmetry (e.g. Bechshøft et al. 2008), measurements of paired characters were taken on the left hand side, where available and possible. Especially for type material, this was not always possible and in order to provide at least some measurement, we also measured photographs taken on the right hand side (noted in Suppl. material 2). Prior to taking photographs and measuring, a Bland-Altman testing procedure (Bland and Altman 1986, Maletti et al. in press) was performed to ensure comparability with former gaugers, from whom data was used for morphometric comparison with the new species (Baur 2015, Lutz 2014, Maletti et al. in press, Suppl. material 8). Ranges of measurements and ratios used in the key were calculated with R 3.0.1 (R Core Team 2013).

We also used the Keyence microscope for making stack-images of qualitative character states. A 3-digit individual code including the notion “Palbi” (e.g. “Palbi 536”) was provided for specimens that were measured, photographed or used as reference specimens for comparison with the newly described species. Specimens already possessing a unique label from former projects (like e.g. “alb”, “Baur” etc.) or that can otherwise be recognised without a doubt (such as holotypes), were not given an additional label.

### Morphometric data

To further test the hypothesis that *P. capito* Baur sp. n. is indeed a separate species, a shape principal component analysis (shape PCA) and a PCA ratio spectrum were applied incorporating 28 of the measurements listed above. The analysis of ratios of body measurements is an important part of morphometric taxonomy in Chalcidoidea, since many cryptic species often differ significantly in body proportions but not in qualitative characters (Baur and Leuenberger 2011). The method has since been known as Multivariate Ratio Analysis (MRA, e.g. László et al. 2013, Gebiola et al. 2017). For further details, we refer to Baur et al. (2014). Due to their higher measurement error, eye length, head length and temple length have been excluded. Analyses of the morphometric data and all graphics were undertaken using R 3.0.1 (R Core Team (2013)) and the package “ggplot2” (Wickham 2016). The best ratios for the separation of *P. capito* Baur sp. n. from *P. albipennis* and *P. cingulipes* were found using the LDA ratio extractor (Baur and Leuenberger 2011, Baur et al. 2014). All measurements and the full R code used for the shape PCA are listed in Suppl. materials 10, 11.

### Rearing

During field trips in summer 2016 in Switzerland and Italy, additional wasp specimens were collected using sweep nets (Noyes 1982) and by collecting infested flower heads of Asteraceae. The latter were transferred to cardboard boxes (length x width x height: 25 x 12 x 4 cm) with a single hole at the front end, where a transparent plastic tube was fixed (following Noyes 1982). The boxes were kept in a climate chamber at 20°C for 9 months. Emerging flies and wasps were attracted to the light and subsequently ended up in the tubes, where they could be easily collected. All animals were then killed with ethyl acetate, preserved in 90% ethanol and stored in a refrigerator (Suppl. materials 6, 7).

## Data resources

Morphometric raw data files, R-scripts used for calculating the shape PCA and ratios, photographs of measured and illustrated characters and species, as well as files listing examined and prepared specimens are deposited at zenodo.org. An interactive version of the Xper3 key is available under http://pteromalus.identificationkey.fr.

## Taxon treatments

### Pteromalus capito

Baur sp. n.

urn:lsid:zoobank.org:act:C581F9C7-6CF5-4EEF-BB64-67530C56BAB2

#### Materials

- **Type status:** Holotype. **Occurrence:** recordedBy: H. Baur; individualCount: 1; sex: female; lifeStage: adult; preparations: dry mounted; **Taxon:** scientificName: Pteromalus capito; kingdom: Animalia; phylum: Arthropoda; class: Insecta; order: Hymenoptera; family: Pteromalidae; genus: Pteromalus; specificEpithet: capito; taxonRank: species; scientificNameAuthorship: Baur; **Location:** continent: Europe; country: Switzerland; locality: Wallis, Champéry, Col de Bretolet; decimalLatitude: 46.143853; decimalLongitude: 6.795059; georeferenceProtocol: label; **Identification:** identifiedBy: H.Baur; dateIdentified: 2017; **Event:** samplingProtocol: sweeping; eventDate: 03/08/2015; habitat: Alpine meadow; **Record Level:** type: Holotype; language: de; institutionID: NMBE; institutionCode: Natural History Museum Bern (NMBE); collectionCode: NMBE_HB_621; basisOfRecord: PreservedSpecimen
- **Type status:** Paratype. **Occurrence:** recordedBy: H. Baur; individualCount: 1; sex: female; lifeStage: adult; preparations: dry mounted; **Taxon:** scientificName: Pteromalus capito; kingdom: Animalia; phylum: Arthropoda; class: Insecta; order: Hymenoptera; family: Pteromalidae; genus: Pteromalus; specificEpithet: capito; taxonRank: species; scientificNameAuthorship: Baur; **Location:** continent: Europe; country: Switzerland; locality: Wallis, Champéry, Col de Bretolet; decimalLatitude: 46.143853; decimalLongitude: 6.795059; georeferenceProtocol: label; **Identification:** identifiedBy: H.Baur; dateIdentified: 2017; **Event:** samplingProtocol: sweeping; eventDate: 08/06/2013; habitat: Alpine meadow; **Record Level:** type: Paratype; language: de; institutionID: NMBE; institutionCode: Natural History Museum Bern (NMBE); collectionCode: NMBE_HB_620; basisOfRecord: PreservedSpecimen
- **Type status:** Paratype. **Occurrence:** recordedBy: H. Baur; individualCount: 1; sex: female; lifeStage: adult; preparations: dry mounted; **Taxon:** scientificName: Pteromalus capito; kingdom: Animalia; phylum: Arthropoda; class: Insecta; order: Hymenoptera; family: Pteromalidae; genus: Pteromalus; specificEpithet: capito; taxonRank: species; scientificNameAuthorship: Baur; **Location:** continent: Europe; country: Switzerland; locality: Wallis, Champéry, Col de Bretolet; decimalLatitude: 46.143445; decimalLongitude: 6.794963; georeferenceProtocol: label; **Identification:** identifiedBy: H.Baur; dateIdentified: 2017; **Event:** samplingProtocol: sweeping; eventDate: 08/06/2013; habitat: Alpine meadow; **Record Level:** type: Paratype; language: de; institutionID: NMBE; institutionCode: Natural History Museum Bern (NMBE); collectionCode: NMBE_HB_624; basisOfRecord: PreservedSpecimen
- **Type status:** Paratype. **Occurrence:** recordedBy: H. Baur; individualCount: 1; sex: female; lifeStage: adult; preparations: dry mounted; **Taxon:** scientificName: Pteromalus capito; kingdom: Animalia; phylum: Arthropoda; class: Insecta; order: Hymenoptera; family: Pteromalidae; genus: Pteromalus; specificEpithet: capito; taxonRank: species; scientificNameAuthorship: Baur; **Location:** continent: Europe; country: Switzerland; locality: Wallis, Champéry, Col de Bretolet; decimalLatitude: 46.143445; decimalLongitude: 6.794963; georeferenceProtocol: label; **Identification:** identifiedBy: H.Baur; dateIdentified: 2017; **Event:** samplingProtocol: sweeping; eventDate: 08/06/2013; habitat: Alpine meadow; **Record Level:** type: Paratype; language: de; institutionID: NMBE; institutionCode: Natural History Museum Bern (NMBE); collectionCode: NMBE_HB_625; basisOfRecord: PreservedSpecimen
- **Type status:** Paratype. **Occurrence:** recordedBy: H. Baur; individualCount: 1; sex: female; lifeStage: adult; preparations: dry mounted; **Taxon:** scientificName: Pteromalus capito; kingdom: Animalia; phylum: Arthropoda; class: Insecta; order: Hymenoptera; family: Pteromalidae; genus: Pteromalus; specificEpithet: capito; taxonRank: species; scientificNameAuthorship: Baur; **Location:** continent: Europe; country: Switzerland; locality: Wallis, Champéry, Col de Bretolet; decimalLatitude: 46.144322; decimalLongitude: 6.794976; georeferenceProtocol: label; **Identification:** identifiedBy: H.Baur; dateIdentified: 2017; **Event:** samplingProtocol: sweeping; eventDate: 08/06/2013; habitat: Alpine meadow; **Record Level:** type: Paratype; language: de; institutionID: NMBE; institutionCode: Natural History Museum Bern (NMBE); collectionCode: NMBE_HB_622; basisOfRecord: PreservedSpecimen
- **Type status:** Paratype. **Occurrence:** recordedBy: H. Baur; individualCount: 1; sex: female; lifeStage: adult; preparations: dry mounted; **Taxon:** scientificName: Pteromalus capito; kingdom: Animalia; phylum: Arthropoda; class: Insecta; order: Hymenoptera; family: Pteromalidae; genus: Pteromalus; specificEpithet: capito; taxonRank: species; scientificNameAuthorship: Baur; **Location:** continent: Europe; country: Switzerland; locality: Wallis, Champéry, Col de Bretolet; decimalLatitude: 46.144322; decimalLongitude: 6.794976; georeferenceProtocol: label; **Identification:** identifiedBy: H.Baur; dateIdentified: 2017; **Event:** samplingProtocol: sweeping; eventDate: 08/06/2013; habitat: Alpine meadow; **Record Level:** type: Paratype; language: de; institutionID: NMBE; institutionCode: Natural History Museum Bern (NMBE); collectionCode: NMBE_HB_623; basisOfRecord: PreservedSpecimen
- **Type status:** Paratype. **Occurrence:** recordedBy: H. Baur; individualCount: 1; sex: female; lifeStage: adult; preparations: dry mounted; **Taxon:** scientificName: Pteromalus capito; kingdom: Animalia; phylum: Arthropoda; class: Insecta; order: Hymenoptera; family: Pteromalidae; genus: Pteromalus; specificEpithet: capito; taxonRank: species; scientificNameAuthorship: Baur; **Location:** continent: Europe; country: Switzerland; locality: Wallis, Champéry, Col de Bretolet; decimalLatitude: 46.143652; decimalLongitude: 6.795199; georeferenceProtocol: label; **Identification:** identifiedBy: H.Baur; dateIdentified: 2017; **Event:** samplingProtocol: sweeping; eventDate: 08/06/2013; habitat: Alpine meadow; **Record Level:** type: Paratype; language: de; institutionID: NMBE; institutionCode: Natural History Museum Bern (NMBE); collectionCode: NMBE_HB_626; basisOfRecord: PreservedSpecimen
- **Type status:** Paratype. **Occurrence:** recordedBy: H. Baur; individualCount: 1; sex: female; lifeStage: adult; preparations: dry mounted; **Taxon:** scientificName: Pteromalus capito; kingdom: Animalia; phylum: Arthropoda; class: Insecta; order: Hymenoptera; family: Pteromalidae; genus: Pteromalus; specificEpithet: capito; taxonRank: species; scientificNameAuthorship: Baur; **Location:** continent: Europe; country: Switzerland; locality: Wallis, Champéry, Col de Bretolet; decimalLatitude: 46.143652; decimalLongitude: 6.795199; georeferenceProtocol: label; **Identification:** identifiedBy: H.Baur; dateIdentified: 2017; **Event:** samplingProtocol: sweeping; eventDate: 08/06/2013; habitat: Alpine meadow; **Record Level:** type: Paratype; language: de; institutionID: NMBE; institutionCode: Natural History Museum Bern (NMBE); collectionCode: NMBE_HB_627; basisOfRecord: PreservedSpecimen
- **Type status:** Paratype. **Occurrence:** recordedBy: H. Baur; individualCount: 1; sex: female; lifeStage: adult; preparations: dry mounted; **Taxon:** scientificName: Pteromalus capito; kingdom: Animalia; phylum: Arthropoda; class: Insecta; order: Hymenoptera; family: Pteromalidae; genus: Pteromalus; specificEpithet: capito; taxonRank: species; scientificNameAuthorship: Baur; **Location:** continent: Europe; country: Switzerland; locality: Wallis, Champéry, Col de Bretolet; decimalLatitude: 46.143882; decimalLongitude: 6.795086; georeferenceProtocol: label; **Identification:** identifiedBy: H.Baur; dateIdentified: 2017; **Event:** samplingProtocol: sweeping; eventDate: 08/06/2013; habitat: Alpine meadow; **Record Level:** type: Paratype; language: de; institutionID: NMBE; institutionCode: Natural History Museum Bern (NMBE); collectionCode: NMBE_HB_628; basisOfRecord: PreservedSpecimen
- **Type status:** Paratype. **Occurrence:** recordedBy: H. Baur; individualCount: 1; sex: female; lifeStage: adult; preparations: dry mounted; **Taxon:** scientificName: Pteromalus capito; kingdom: Animalia; phylum: Arthropoda; class: Insecta; order: Hymenoptera; family: Pteromalidae; genus: Pteromalus; specificEpithet: capito; taxonRank: species; scientificNameAuthorship: Baur; **Location:** continent: Europe; country: Switzerland; locality: Wallis, Champéry, Col de Bretolet; decimalLatitude: 46.143882; decimalLongitude: 6.795086; georeferenceProtocol: label; **Identification:** identifiedBy: H.Baur; dateIdentified: 2017; **Event:** samplingProtocol: sweeping; eventDate: 08/06/2013; habitat: Alpine meadow; **Record Level:** type: Paratype; language: de; institutionID: NMBE; institutionCode: Natural History Museum Bern (NMBE); collectionCode: NMBE_HB_629; basisOfRecord: PreservedSpecimen
- **Type status:** Paratype. **Occurrence:** recordedBy: H. Baur; individualCount: 1; sex: female; lifeStage: adult; preparations: dry mounted; **Taxon:** scientificName: Pteromalus capito; kingdom: Animalia; phylum: Arthropoda; class: Insecta; order: Hymenoptera; family: Pteromalidae; genus: Pteromalus; specificEpithet: capito; taxonRank: species; scientificNameAuthorship: Baur; **Location:** continent: Europe; country: Switzerland; locality: Wallis, Champéry, Col de Bretolet; decimalLatitude: 46.144121; decimalLongitude: 6.794942; georeferenceProtocol: label; **Identification:** identifiedBy: H.Baur; dateIdentified: 2017; **Event:** samplingProtocol: sweeping; eventDate: 08/06/2013; habitat: Alpine meadow; **Record Level:** type: Paratype; language: de; institutionID: NMBE; institutionCode: Natural History Museum Bern (NMBE); collectionCode: NMBE_HB_630; basisOfRecord: PreservedSpecimen
- **Type status:** Paratype. **Occurrence:** recordedBy: H. Baur; individualCount: 1; sex: female; lifeStage: adult; preparations: dry mounted; **Taxon:** scientificName: Pteromalus capito; kingdom: Animalia; phylum: Arthropoda; class: Insecta; order: Hymenoptera; family: Pteromalidae; genus: Pteromalus; specificEpithet: capito; taxonRank: species; scientificNameAuthorship: Baur; **Location:** continent: Europe; country: Switzerland; locality: Wallis, Champéry, Col de Bretolet; decimalLatitude: 46.144121; decimalLongitude: 6.794942; georeferenceProtocol: label; **Identification:** identifiedBy: H.Baur; dateIdentified: 2017; **Event:** samplingProtocol: sweeping; eventDate: 08/06/2013; habitat: Alpine meadow; **Record Level:** type: Paratype; language: de; institutionID: NMBE; institutionCode: Natural History Museum Bern (NMBE); collectionCode: NMBE_HB_631; basisOfRecord: PreservedSpecimen
- **Type status:** Paratype. **Occurrence:** recordedBy: H. Baur; individualCount: 1; sex: female; lifeStage: adult; preparations: dry mounted; **Taxon:** scientificName: Pteromalus capito; kingdom: Animalia; phylum: Arthropoda; class: Insecta; order: Hymenoptera; family: Pteromalidae; genus: Pteromalus; specificEpithet: capito; taxonRank: species; scientificNameAuthorship: Baur; **Location:** continent: Europe; country: Switzerland; locality: Wallis, Champéry, Col de Bretolet; decimalLatitude: 46.14371; decimalLongitude: 6.795148; georeferenceProtocol: label; **Identification:** identifiedBy: H.Baur; dateIdentified: 2017; **Event:** samplingProtocol: sweeping; eventDate: 08/06/2013; habitat: Alpine meadow; **Record Level:** type: Paratype; language: de; institutionID: NMBE; institutionCode: Natural History Museum Bern (NMBE); collectionCode: NMBE_HB_632; basisOfRecord: PreservedSpecimen
- **Type status:** Paratype. **Occurrence:** recordedBy: H. Baur; individualCount: 1; sex: female; lifeStage: adult; preparations: dry mounted; **Taxon:** scientificName: Pteromalus capito; kingdom: Animalia; phylum: Arthropoda; class: Insecta; order: Hymenoptera; family: Pteromalidae; genus: Pteromalus; specificEpithet: capito; taxonRank: species; scientificNameAuthorship: Baur; **Location:** continent: Europe; country: Switzerland; locality: Wallis, Champéry, Col de Bretolet; decimalLatitude: 46.14371; decimalLongitude: 6.795148; georeferenceProtocol: label; **Identification:** identifiedBy: H.Baur; dateIdentified: 2017; **Event:** samplingProtocol: sweeping; eventDate: 08/06/2013; habitat: Alpine meadow; **Record Level:** type: Paratype; language: de; institutionID: NMBE; institutionCode: Natural History Museum Bern (NMBE); collectionCode: NMBE_HB_633; basisOfRecord: PreservedSpecimen
- **Type status:** Paratype. **Occurrence:** recordedBy: H. Baur; individualCount: 1; sex: female; lifeStage: adult; preparations: dry mounted; **Taxon:** scientificName: Pteromalus capito; kingdom: Animalia; phylum: Arthropoda; class: Insecta; order: Hymenoptera; family: Pteromalidae; genus: Pteromalus; specificEpithet: capito; taxonRank: species; scientificNameAuthorship: Baur; **Location:** continent: Europe; country: Switzerland; locality: Ticino, Faido, Garlengo; decimalLatitude: 46.456363; decimalLongitude: 8.796262; georeferenceProtocol: label; **Identification:** identifiedBy: H.Baur; dateIdentified: 2017; **Event:** samplingProtocol: sweeping; eventDate: 08/06/2013; habitat: Marsh area; **Record Level:** type: Paratype; language: de; institutionID: NMBE; institutionCode: Natural History Museum Bern (NMBE); collectionCode: NMBE_HB_634; basisOfRecord: PreservedSpecimen
- **Type status:** Paratype. **Occurrence:** recordedBy: H. Baur; individualCount: 1; sex: female; lifeStage: adult; preparations: dry mounted; **Taxon:** scientificName: Pteromalus capito; kingdom: Animalia; phylum: Arthropoda; class: Insecta; order: Hymenoptera; family: Pteromalidae; genus: Pteromalus; specificEpithet: capito; taxonRank: species; scientificNameAuthorship: Baur; **Location:** continent: Europe; country: Switzerland; locality: Wallis, Champéry, Col de Bretolet; decimalLatitude: 46.142351; decimalLongitude: 6.795546; georeferenceProtocol: label; **Identification:** identifiedBy: H.Baur; dateIdentified: 2017; **Event:** samplingProtocol: sweeping; eventDate: 30/07/2003; habitat: Alpine meadow; **Record Level:** type: Paratype; language: de; institutionID: NMBE; institutionCode: Natural History Museum Bern (NMBE); collectionCode: NMBE_HB_694; basisOfRecord: PreservedSpecimen
- **Type status:** Paratype. **Occurrence:** recordedBy: H. Baur; individualCount: 1; sex: female; lifeStage: adult; preparations: dry mounted; **Taxon:** scientificName: Pteromalus capito; kingdom: Animalia; phylum: Arthropoda; class: Insecta; order: Hymenoptera; family: Pteromalidae; genus: Pteromalus; specificEpithet: capito; taxonRank: species; scientificNameAuthorship: Baur; **Location:** continent: Europe; country: Switzerland; locality: Wallis, Champéry, Col de Bretolet; decimalLatitude: 46.142351; decimalLongitude: 6.795546; georeferenceProtocol: label; **Identification:** identifiedBy: H.Baur; dateIdentified: 2017; **Event:** samplingProtocol: sweeping; eventDate: 30/07/2003; habitat: Alpine meadow; **Record Level:** type: Paratype; language: de; institutionID: NMBE; institutionCode: Natural History Museum Bern (NMBE); collectionCode: NMBE_HB_695; basisOfRecord: PreservedSpecimen
- **Type status:** Paratype. **Occurrence:** recordedBy: H. Baur; individualCount: 1; sex: female; lifeStage: adult; preparations: dry mounted; **Taxon:** scientificName: Pteromalus capito; kingdom: Animalia; phylum: Arthropoda; class: Insecta; order: Hymenoptera; family: Pteromalidae; genus: Pteromalus; specificEpithet: capito; taxonRank: species; scientificNameAuthorship: Baur; **Location:** continent: Europe; country: Switzerland; locality: Wallis, Champéry, Col de Bretolet; decimalLatitude: 46.142351; decimalLongitude: 6.795546; georeferenceProtocol: label; **Identification:** identifiedBy: H.Baur; dateIdentified: 2017; **Event:** samplingProtocol: sweeping; eventDate: 30/07/2003; habitat: Alpine meadow; **Record Level:** type: Paratype; language: de; institutionID: NMBE; institutionCode: Natural History Museum Bern (NMBE); collectionCode: NMBE_HB_696; basisOfRecord: PreservedSpecimen

#### Description

**Female**: *Colour*: Head and mesosoma: green to blue-green with metallic lustre; setae on head and mesosoma: whitish, inconspicuous; tegula: testaceous; setae on callus of popodeum: whitish. Scape: fuscous with basal third to half testaceous; pedicel: fuscous; flagellum: brown to black. Fore wing: hyaline; fore wing venation: testaceous; setae on fore wing: whitish except for setae on the lower surface of the costal cell and for some setae along the distal part of the marginal vein which are fuscous; hind wing: hyaline. Procoxa: green-blue; meso- and metacoxa: infuscate with metallic tinge; trochanters: infuscate, testaceous at tips; profemur: infuscate, testaceous in apical sixth; mesofemur: infuscate, testaceous in apical and basal tips; metafemur: infuscate with metallic tinge, testaceous in apical tenth; pro- and mesotibiae: testaceous, medially slightly infuscate; metatibia: infuscate, basal fifth and apical third testaceous; protarsus: testaceous with distal segments infuscate; meso- and metatarsus: testaceous; pretarsi: infuscate. Petiole: dark green with purplish tinge; gaster: dark blue-green with metallic lustre; gastral terga: one to five green to blue. *Sculpture*: Head in frontal view: finely reticulate with moderately high septa; clypeus: striate; area between clypeus and malar sulcus: finely reticulate. Mesoscutum: finely reticulate, meshes moderately high, areoles slightly enlarged medially in posterior part of sclerite; scutellum: reticulate, meshes about as strong and coarse as on posterior part of mesoscutum; frenum: reticulate, meshes mostly similar in size to those on scutellum; axilla: reticulate, about as strongly as on central part of scutellum; prepectus upper triangular area: weakly reticulate to almost smooth; upper mesepimeron: smooth to alutaceous; upper mesepisternum: reticulate, about as strongly as on mesoscutum; metapleuron: finely reticulate, less strongly than on mesepisternum. Procoxa: alutaceous to finely reticulate; mesocoxa: finely alutaceous; metacoxa: finely reticulate. Median area of propodeum: finely reticulate, less strongly than on mesoscutum, with longitudinal ridges; inner corner of anterior plica: with a large depression, alutaceous; nucha: finely reticulate, meshes transverse, less strong than on median area of propodeum; callus of propodeum: finely reticulate; paraspiracular sulcus: smooth with few transverse costulae. Petiole in dorsal view: smooth; gastral terga: smooth and shining, second to fifth tergum anteriorly alutaceous, sixth and syntergum entirely alutaceous. *Shape and structure*: Head in frontal view: subtrapezoid; gena in frontal view: slighty buccate; temple in dorsal view: obtuse; forming an angle with occiput of: 110 degrees; occipital carina: absent; torulus position with respect to lower ocular line: distinctly above; lower face in lateral view: rather flat; receding with respect to upper face: weakly; forming an angle of 35 degrees; scrobe: narrow, moderately deep; malar sulcus: superficial, but traceable; clypeus anterior margin: narrowly and shallowly emarginate, without a depression above emarginate edge; gena near mouth: terete; tentorial pit: indistinct; mouth extension: not conspicuously enlarged; mandibular formula: 3-4. Antenna: Antennal formula: 11263; scape reaching: middle of anterior ocellus; flagellum: weakly clavate; first anellus: strongly transverse; second anellus: strongly transverse; first funicular segment: slightly conical; setae on flagellum: thickly clothed with subdecumbent setae, length of setae at most half as long as breadth of flagellar segments; number of rows of longitudinal sensilla on first funicular segment: 2; on sixth: 1-2. Mesosoma in lateral view: moderately bent; propodeum in lateral view sloping with respect to dorsal plane of mesoscutum and scutellum at an angle of 40 degrees; pronotum breadth with respect to mesoscutum breadth: distinctly narrower; pronotum collar: horizontal, well defined; its length with respect to mesoscutum length: slightly more than one sixth; its anterior margin: slightly elevated ridge, sometimes medially carinate; pronotum posterior margin: finely reticulate, shiny; notauli: superficial; reaching: two thirds along mesoscutum; scutellum in lateral view: moderately convex, slightly depressed around the middle; scutellum in posterior view: convex; scutellum posterior margin projection: level of anterior margin of dorsellum; scutellum posterior margin in posterior view: narrowly emarginate in the middle; frenal line: finely indicated, especially on sides; prepectus upper triangular area: separated by an indistinct oblique carina; upper mesepimeron: strongly narrowing below, not reaching base of mesopleuron; propodeum anterior plica: strong, bent inwards posteriorly to form an elevated costula; posterior plica: present, joining anterior plica; orientation of posterior plica: strongly converging; median carina of propodeum: present, often bifurcating from base of propodeum; nucha: lunate strip, clearly delimited from median area by an edge; spiracle: oval, almost touching anterior margin of propodeum; size: moderate; callus pilosity: sparsely pilose; paraspiracular sulcus: with large and moderately deep fovea slightly offset from spiracle, bluntly reflexed at level of posterior edge of fovea. Fore wing: Fore wing apex with respect to apex of gaster when folded back: slightly exceeding; basal cell number of setae: bare; basal setal line: incomplete; with 0 setae; cubital setal line: incomplete; with 0 setae; costal cell pilosity on dorsal side: bare; costal setal line: widely interrupted medially; speculum on dorsal side: bare, extending or almost extending to stigmal vein, widely open below; wing disc: sparsely pilose; marginal setae: missing; stigma: oval to subrhombical, small; uncus: short. Femora: moderately slender; metatibia: gradually widening towards apex; metacoxa pilosity dorsal: bare. Petiole in dorsal view: conical; in ventral view: open; gaster in dorsal view: elongate, acuminate; gastral terga: convex, sometimes weakly sunken; posterior margin of first gastral tergum: entire; first gastral tergum reaching: one fifth of gaster; tip of hypopygium reaching about half of gaster; ovipositor sheath: distinctly protruding. *Length and body ratios*: Body length: 2.8—4.0 mm; mesoscutum breadth: 635—1002 μm. Head breadth to head height: 1.30—1.45; head breadth to length: 1.89—2.25; head breadth to mesoscutum breadth: 1.02—1.15; upper face height to head height: 0.56—0.61; POL to OOL: 1.26—1.52; eye height to breadth: 1.34—1.52; eye distance to height: 1.38—1.52; temple length to eye height: 0.21—0.42; malar space to eye height: 0.46—0.54. Antenna length to head breadth: 0.86—0.94; scape length to eye height: 0.72—0.80; pedicel length to breadth: 1.33—1.94. Mesosoma length to mesoscutum breadth: 1.28—1.50; mesoscutum breadth to length: 1.43—1.87; mesoscutum length to scutellum length: 1.05—1.28; propodeum length to scutellum length: 0.29—0.42; plica distance to propodeum length: 1.90—3.05. Fore wing length to breadth: 2.15—2.36; marginal vein to stigma vein length: 1.44—1.80. Metafemur length to breadth: 3.46—3.76. Gaster length to breadth: 1.93—2.39; gaster length to mesosoma length: 1.43—1.66.

#### Diagnosis

The female of *P. capito* Baur sp. n. keys out in Graham (1969) via couplets 1, 2, 7—9, 11, 12, 14—19, 21, 22, 24 to *P. caudiger* (alternatively couplets 22, 24, 25 to *P. albipennis*), where it fits neither description. The species belongs to a group of species with an interrupted costal setal line (i.e. to the *Pteromalus albipennis* group sensu Graham 1969). In this group, it is most similar to *P. albipennis*. It is distinguished from this and all other species by the following combination of characters: Clypeus emargination narrow and smooth, distal part of scape fuscous (Fig. 5c), head posterior to eye relatively broad (Fig. 4a), sculpture of middle part of propodeum reticulate (Fig. 4d), reticulation on frenum median area coarser than on scutellum median area, pronotal collar one seventh to one sixth the length of mesoscutum, often slightly elevated anteriorly (Fig. 4c), setae on fore wing almost exclusively pale (Fig. 4b), proximal half of metatibia with fuscous infumation (Fig. 5b), metacoxa only apically with setae, paraspiracular inclination blunt (Fig. 3b), area below propodeal foramen with pit absent and fissure present (Fig. 2c), ratio of antenna length to OOL <5.85, ratio of eye distance to POL >2.73. Below, the most important differences are given for those species with which *P. capito* Baur sp. n. might be most easily confused (character states, where the other species differ from *P. capito* Baur sp. n., are underlined).

*P. achillei*: Clypeus emargination narrow and smooth or foveate, distal part of scape fuscous or testaceous, head posterior to eye relatively narrower, sculpture of middle part of propodeum reticulate, transversely rugulose or smooth, reticulation on frenum median area coarser than or as coarse as on scutellum median area, pronotal collar one seventh to one sixth the length of mesoscutum, not elevated anteriorly, setae on fore wing fuscous, proximal half of metatibia yellow or with fuscous infumation, metacoxa only apically with setae, paraspiracular inclination sharp or blunt, area below propodeal foramen with pit present or absent, fissure absent.

*P. albipennis*: Clypeus emargination narrow and foveate, distal part of scape fuscous (Fig. 8c), head posterior to eye relatively narrower (Fig. 7a), sculpture of middle part of propodeum reticulate or transversely rugulose (Fig. 7d), reticulation on frenum median area coarser than or as coarse as on scutellum median area, pronotal collar one seventh to one sixth the length of mesoscutum, not elevated anteriorly (Fig. 7c), setae on fore wing pale or fuscous (Fig. 7b), proximal half of metatibia yellow or with fuscous infumation (Fig. 8b), metacoxa only apically with setae (Fig. 8b), paraspiracular inclination sharp (Fig. 3a), area below propodeal foramen with pit present, fissure present or absent, ratio of antenna length to OOL >5.85, ratio of eye distance to POL <2.73.

*P. caudiger*: Clypeus emargination narrow and foveate, distal part of scape fuscous, head posterior to eye relatively narrower, sculpture of middle part of propodeum reticulate or transversely rugulose, reticulation on frenum median area coarser than on scutellum median area, pronotal collar one seventh to one sixth the length of mesoscutum, usually not elevated anteriorly, setae on fore wing fuscous, proximal half of metatibia yellow or with fuscous infumation, metacoxa only apically with setae, paraspiracular inclination sharp (Fig. 3a), area below propodeal foramen with pit present and fissure absent (Fig. 2a).

*P. cingulipes*: Clypeus emargination narrow and smooth or foveate, distal part of scape fuscous or testaceous, head posterior to eye relatively narrower, sculpture of middle part of propodeum reticulate or transversely rugulose, reticulation on frenum median area coarser than or as coarse as on scutellum median area, pronotal collar one seventh to one sixth the length of mesoscutum, usually not elevated anteriorly, setae on fore wing pale, proximal half of metatibia with almost black infumation, metacoxa only apically with setae, paraspiracular inclination sharp (Fig. 3a), area below propodeal foramen with pit present or absent, fissure absent, ratio of eye distance to POL <2.73.

*P. eudecipiens*: Clypeus emargination narrow or wide and smooth, distal part of scape fuscous or testaceous, head posterior to eye relatively narrower, sculpture of middle part of propodeum reticulate or transversely rugulose, reticulation on frenum median area coarser than or as coarse as on scutellum median area, pronotal collar one seventh to one sixth the length of mesoscutum, not elevated anteriorly, setae on fore wing fuscous, proximal half of metatibia yellow or with fuscous infumation, metacoxa only apically with setae, paraspiracular inclination sharp (Fig. 3a), area below propodeal foramen with pit present and fissure present or absent.

*P. intermedius*: Clypeus emargination wide and smooth, distal part of scape fuscous or testaceous, head posterior to eye relatively narrower, sculpture of middle part of propodeum reticulate or transversely rugulose, reticulation on frenum median area coarser than or as coarse as on scutellum median area, pronotal collar one fifth the length of mesoscutum, mostly elevated anteriorly, setae on fore wing fuscous, proximal half of metatibia yellow or with fuscous infumation, metacoxa only apically with setae, paraspiracular inclination blunt (Fig. 3b), area below propodeal foramen with pit absent, fissure present (Fig. 2c).

*P. patro*: Clypeus emargination narrow and foveate, distal part of scape fuscous, head posterior to eye relatively narrower, sculpture of middle part of propodeum transversely rugulose, reticulation on frenum median area coarser than on scutellum median area, pronotal collar one seventh to one sixth the length of mesoscutum, not elevated anteriorly, setae on fore wing fuscous, proximal half of metatibia yellow or with fuscous infumation, metacoxa only apically with setae, paraspiracular inclination sharp (Fig. 3a), area below propodeal foramen with pit present, fissure absent (Fig. 2a).

*P. temporalis*: Clypeus emargination wide and smooth, distal part of scape testaceous, head posterior to eye relatively narrower, sculpture of middle part of propodeum reticulate or transversely rugulose, reticulation on frenum median area coarser than or as coarse as on scutellum median area, pronotal collar one fifth the length of mesoscutum, usually not elevated anteriorly, setae on fore wing fuscous, proximal half of metatibia yellow, metacoxa with a few short setae also basally, paraspiracular inclination blunt (Fig. 3b), area below propodeal foramen with pit absent, fissure absent (Fig. 2b).

#### Etymology

The species epithet “capito” refers to the relatively massive head of the species in comparison to its morphologically closest relatives. The word derives from Latin “capito” meaning “one that has a large head, big-headed”. It is a noun that does not change with gender.

#### Biology

*Pteromalus capito* Baur sp. n. is a parasitoid of larvae of Tephritidae (Diptera). Reared specimens emerged from *Crepis pyrenaica* (L.) Moench (coll. 07.07.2016) together with *Tephritis crepidis* Hendel, from *Crepis paludosa* (L.) Moench (coll. 21.07.2016) together with *Tephritis conura* (Loew), *Campiglossa cf. guttella* (Rond.) and *Campiglossa cf. producta* (Loew) and from *Centaurea jacea* L. s.l. (coll. 27.07.2016) together with *Chaetoriella jaceae* (Rob.-Desv.) and *Acanthiophilus helianthi* (Rossi) (Suppl. materials 6, 7).

#### Morphometric data

Fig. 9a shows the scatterplot of the PCA for *Pteromalus albipennis*, *P. capito* Baur sp. n. and *P. cingulipes* (28 measurements). *P. capito* Baur sp. n. is clearly separated from *P. cingulipes* along PC1 but shows some overlap with *P. albipennis* along both axes. A closer look at the explanatory power of the single principal components reveals that PC1 only explains 20.8%, which shows that the differences between *P. cingulipes* and a group composed of *P. capito* Baur sp. n. and *P. albipennis* are marginal. In total, both axes explain 38.6% of the variation, indicating a close resemblance of all three species. Fig. 9b shows the results of the PCA with *P. cingulipes* excluded. The data clouds of *P. capito* Baur sp. n. and *P. albipennis* are now also completely separated along PC1, although the difference is relatively small with 26.2% of the variation explained. PC2 explains 20.4% and together they explain 46.6% of the variation. Allometric effects can be excluded, since the two groups do not lie on a common allometric axis (Fig. 9c). The PCA ratio spectrum of Shape PC1 shows several characters which dominate the separation: ool.l, gst.b, clv.b, stv.l and pol.l (Fig. 9d). These are the characters positioned far apart from each other and therefore contribute most to the variation explained by PC1. All other characters are clustered together in the middle of the axis and have thus only a marginal influence on the separation of the two species. The best ratios to separate *P. capito* Baur sp. n. from its closest relatives are shown in Table 3.

The measurements for all species are listed in Suppl. materials 2, 3, 4, 9.

## Analysis

### New morphological characters

No study so far has examined the area of the metasternum in Pteromalidae for distinctive characters although it seems quite promising. In Fig. 2a, b, c, we can see the propodeal foramen flanked by the foramen of the metacoxa. In between, one pair of fine wrinkles delimits the inner area of the metasternum from the paraspiracular inclination. These wrinkles may be complemented ventrally by additional wrinkles in some species, providing a first new distinctive character. Below the propodeal foramen, a fissure is present in some species, dividing the core area of the metasternum vertically into two parts. An alternative state is present, when some sort of broad impression bulges the core area inwards. However, it is also possible for species to lack both of these states and simply show a flat and smooth surface. As an additional character, the shape of the propodeal foramen can be defined as being either oval or round. Ventrally of the metacoxal foramen, a horizontal set of wrinkles concludes the metasternum. For better visibility, the mesocoxa were removed as well.

The preparation of translucent slide material of the back of the head yielded no distinguishing characters. Fig. 6 shows the heads of *P. achillei* and *P. cingulipes* in posterior view. When comparing *P. capito* Baur sp. n. to closely related species, such as *P. albipennis*, it was obvious that the paraspiracular inclination differed. By grinding the specimens laterally to the level of the spiracle, the tegula, lateral panel of axilla, prepectus, acropleuron, upper mesepimeron and metapleuron of the respective side were removed almost completely. Peripheral structures like the lateral lobe of mesoscutum, axilla and lower mesepimeron were at least truncated. For better visibility of the area of interest, the metacoxa, wings and gaster were removed before grinding. Treating the specimens with a microtome clarified the differences in the paraspiracular inclination. The edge of the propodeum describes either a sharp bend towards the metasternum or else is gently rounded (Fig. 3).

## Discussion

### Interactive identification key

The development of software to analyse large amounts of descriptive data allowed the creation of new taxonomic keys, which led to a facilitated identification process. As an example, the identification of *P. albipennis* needs 16 steps in Graham (1969), whereas with our interactive key only 6 steps are necessary in the best case. The number of steps strongly depends on the selection of the characters, which in our key are ranked according to their relevance for the *P. albipennis* species group. This means that this identification path is optimised and that species from other groups may take longer than in Graham (1969). Although Graham’s key comes with a lot of detailed drawings, only a fraction of the characters could be illustrated. This makes it often difficult to understand the differences between two states. Our key is completely illustrated and comes with 585 pictures for all taxa, characters and states. These pictures are directly displayed with every object and therefore easily accessible during the entire identification process. In addition, every taxon is portrayed with its complete diagnosis and a set of pictures illustrating most of the characters of the key. Keys created in Xper3 offer the possibility to share the database with co-workers, who can bring in their latest results. Depending on the rights the owner grants them, they are able to only read or edit the database. This means that they can add new taxa and descriptors, edit existing taxa/descriptors or upload additional illustrations. Constant actualisation is therefore assured. Any changes in the database will not affect the permanent URL, which means that everybody having access to the link will always be working with the latest version. Xper3 comes with the ability to export the database in SDD or CSV files, which can also be processed by other software. Together with the independence from platforms and operating systems, this software allows transfer and editing of data also via alternative ways.The treatment of quantitative characters like measurements and ratios by Xper3 in a non-discrete way is not completely satisfying. It is impossible for an identification key to cover the complete range of variation of a character and therefore users will always have to deal with specimens exceeding the range. Especially if only a few specimens are available for measurement, indicated ranges are not very indicative (see Suppl. material 1). However, for practical reasons and to give it a try, we mostly relinquished making discrete groups, except for some measurements which separate a few closely related species (e.g. see Table 3). The discrete nature of most taxa is nonetheless ensured with the qualitative characters alone.

It must be pointed out that the long term storage of the descriptive data and the pictures as well as the future access to the original database is a major concern. We simply do not have the experience of storing digital data over longer time periods (>20 years) ([Bibr B3769373][Bibr B3770058]). In order to enable future generations to access our key and the data underlying it, we deposited all raw files and the original TIF-pictures on several data repository sites. Since Dropbox, where the pictures linked to the key are hosted, is a commercial provider and is not designed and obliged to ascertain long-term access to scientific data, this way seems practical to us with the current state of knowledge. To enable access to the original database with the right to edit, it would be best to designate a successor, who carries on our work. However, it is not clear if such a person may be found in time. Therefore, we arranged with the team of Xper3 that, if somebody wishes to get writing permission to take care of our database after the corresponding author or his successor has retired, he or she can contact the Xper3 team, which will grant the access. However, it must be clear that the corresponding author must be contacted first if still possible.

### Cryptic diversity in the *P. albipennis* group

It is surprising that, even in one of the taxonomically best studied areas like Central Europe, new species can be found. *Pteromalus capito* Baur sp. n. clearly belongs to the *P. albipennis* species group showing all its distinctive characters (see above). In the recent past, some new species have been described for this species group (Janzon 1984) which later turned out to be just forms of existing species (Lutz 2014). However, the morphometric and morphological data both support the status of *P. capito* Baur sp. n. as a distinct new species, although cryptic in appearance. The shape PCA separates it completely from *P. albipennis* and *P. cingulipes*, its morphologically closest relatives. The ratios with the greatest distinctive power (antenna length to OOL and eye distance to POL) provide not only a sound but also easily measurable means for identification. The broad head (in lateral view) separates it morphologically from all other species of the *P. albipennis* species group. Some *P. albipennis* specimens may show a similarly pale colouration of their wing setae which can be misleading. However, the shape of the paraspiracular inclination, although not blunt exclusively for *P. capito* Baur sp. n. in the species group, in combination with the other characters, is a sound guide to a reliable identification. These qualitative characters have proven to be very consistent within the species, therefore providing good support. Only recently (Maletti et al. in press), *P. capito* Baur sp. n. was included in a study using DNA barcoding. It could be separated very clearly from all other species. These 5 specimens are also part of our morphologic analysis (see above). Our rearing conditions suggest that *P. capito* Baur sp. n. develops as a parasitoid of larvae of Tephritidae (Diptera). Its host range and therefore also its plant association do not significantly differ from other closely related species. From every sample with *Pteromalus capito* Baur sp. n., *P. albipennis* emerged as well. Currently, only *Tephritis crepidis* Hendel can be confirmed as host because it is the only host species with which *P. capito* Baur sp. n. emerged exclusively. However, it is very likely that the other species constitute hosts, too. In addition, we can confirm that *Pteromalus brachygaster* and *P. scandiae*, which were newly described by Graham (1969) and, according to his key, are morphologically very similar to *Pteromalus tibiellus*, are in fact original species. This holds true for *P. conformis* as well, which appears very similar to *Pteromalus inclytus* Förster. These taxa have not been taxonomically reviewed since their original description.

### New morphological characters

The paraspiracular inclination has been shown to be very consistent within species and has therefore proved to be a valuable means for distinguishing especially *P. capito* Baur sp.n. and *P. albipennis* but also other species. It is not absolutely necessary to grind the specimens with a microtome when studying this character. With some experience and a good light microscope, it is possible to spot the differences without preparation. Grinding specimens was merely used to take good pictures illustrating the differences, which was otherwise impossible with the available equipment. Thirteen species showed a sharp edge of the paraspiracular inclination, of which all belong to the *P. albipennis* species group, in comparison to 30 species with a blunt edge (not examined for two species). As far as we know, there have been no comments in the scientific literature mentioning this structure in Pteromalidae. Studying of the area of the metasternum astonishingly revealed a whole bunch of distinctive characters. The wrinkles connecting the propodeal with the metacoxal foramen may show some sort of stabilising function of the metasternum. We assume that the impression below the propodeal foramen is linked to gaster size or ovipositor length, since species with a large gaster tend to have such an impression more often. The presence or absence of a fissure vertically below the propodeal foramen may be linked to developmental processes. The shape of the propodeal foramen, although consistent in most species, may only be an artefact of a small sample size, since we did not have the possibility to dissect a large quantity of specimens for each species. Therefore, this character may need further investigation. The metasternum has also attracted the interest of other researchers in the field of Pteromalidae. Krogmann and Burks (2009) included a brief characterisation of the metasternum in their description of *Doddifoenus wallacei* sp. n. Gibson (2015) gave the relative length of the metasternum and compared the position of the base of the mesocoxa in relation to the base of the metacoxa in his revision of the *Notanisus oulmesiensis* species group. However, the most extensive work so far on this character has been done by Grissell (1995) in his World Catalog for Toryminae species (Chalcidoidea: Torymidae). He studied the metasternum using drawings and scanning electron micrographs and worked out different character states to separate the genera. Together with our work, this raises the question whether the metasternum also provides useful taxonomical characters for other groups of Chalcidoidea.The examination of the back of the head yielded no useful characters here. The area shows hardly any tangible structures besides the occipital foramen. Since *Pteromalus* sp. live on very similar food sources (pollen, nectar), their mouthparts also do not differ consistently. However, Lotfalizadeh et al. (2007) have demonstrated that the back of the head shows taxonomically important characters for Eurytominae (Chalcidoidea: Eurytomidae).

## Supplementary Material

Supplementary material 1 Labelled and measured specimens Data type: list
Brief description: All labelled and measured specimens relevant for this work are listed with their sample date, location, deposition, manipulations etc.
File: oo_153268.csv Klimmek, Fabian & Baur, Hannes

Supplementary material 2 Measurement data Data type: measurements
Brief description: Body measurements for every single individual used for the key. This file includes 15 measurements and 8 ratios.
File: oo_153269.csv Klimmek, Fabian & Baur, Hannes

Supplementary material 3 Body measurements: ranges Data type: Min & max values
Brief description: This file contains the Min and Max values for each species included in the key for the body measurements.
File: oo_153270.csv Klimmek, Fabian & Baur, Hannes

Supplementary material 4 Measurement ratios: ranges Data type: Min and Max values
Brief description: This file contains the Min and Max values for every species in the key for each of the 8 calculated ratios.
File: oo_153271.csv Klimmek, Fabian & Baur, Hannes

Supplementary material 5 Specimens prepared for microtomy Data type: list
Brief description: This files lists all specimens that were treated with the microtome for the examination of the paraspiracular inclination including information of taxon, deposition, sex, sampling data, sampling location etc.
File: oo_153272.csv Klimmek, Fabian & Baur, Hannes

Supplementary material 6 Rearings overview Data type: list
Brief description: This file lists all flower head samples that have been collected during summer 2016 and from which Chalcidoids were reared, including information on sampling date, sampling location, plant species, number and taxon of insects hatched etc.
File: oo_153274.csv Maletti, Sina & Klimmek, Fabian & Baur, Hannes

Supplementary material 7 Rearings species list Data type: species list
Brief description: This file lists all species belonging to Chalcidoidea or Tephritidae which hatched from the collected flower heads.
File: oo_153275.csv Maletti, Sina & Klimmek, Fabian & Baur, Hannes

Supplementary material 8 Bland-Altman training data Data type: morphometric measurements
Brief description: This file contains all measurements (also from former gaugers) used by the authors for the Bland-Altman training, which was performed prior to measuring specimens relevant to the key.
File: oo_153281.csv Maletti, Sina & Schilling, Franziska & Wittmer, Cindy & Klimmek, Fabian & Baur, Hannes

Supplementary material 9 *Pteromalus capito* Baur sp. n. body measurements and ratios: ranges Data type: measurement data
Brief description: This files lists all body measurements, ratios and their Min and Max values for each specimen of *P. capito* Baur sp. n. used for the Principal Component Analysis and the key.
File: oo_153287.csv Klimmek, Fabian & Baur, Hannes

Supplementary material 10 Measurement data for Pteromalus capito Baur sp. n. and reference species Data type: measurement data
Brief description: This file contains all relevant data for the Principal Component Analysis and for the creation of the plots used in this paper.
File: oo_153288.csv Klimmek, Fabian & Baur, Hannes

Supplementary material 11 Pteromalus capito Baur sp. n. Principal component analysis Data type: R file
Brief description: This R file contains the code for the Principal Component Analysis and the creation of the plots used in this work.
File: oo_153290.R Klimmek, Fabian & Baur, Hannes

XML Treatment for Pteromalus capito

## Figures and Tables

**Figure 2a.**
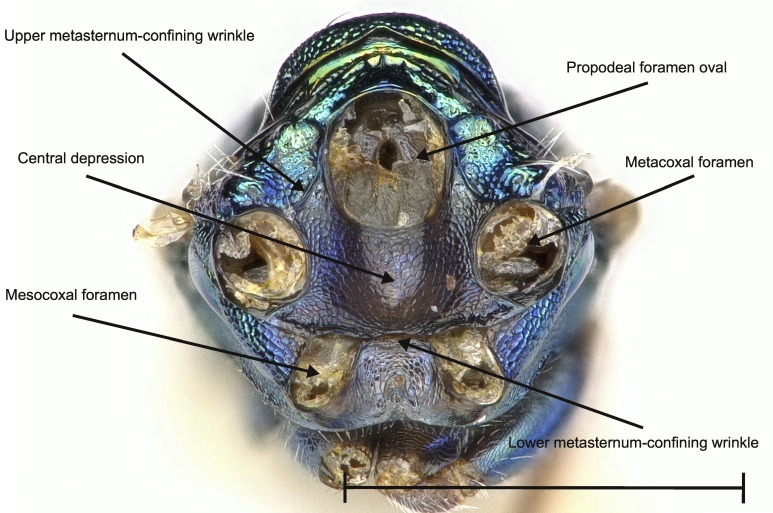
*Pteromalus caudiger*.

**Figure 2b.**
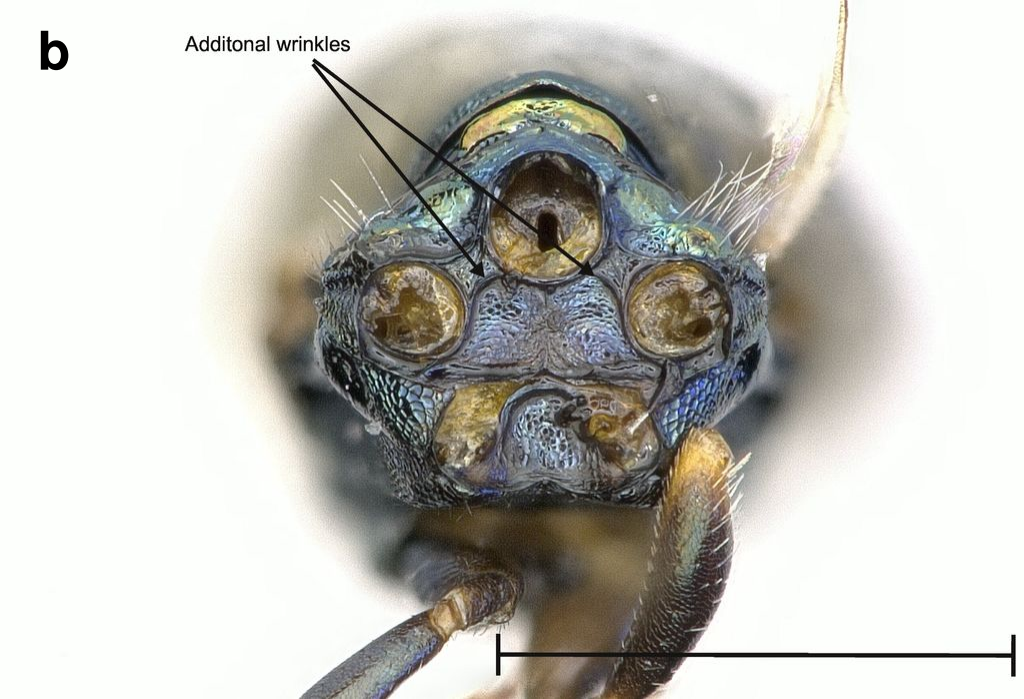
*Pteromalus inclytus.*

**Figure 2c.**
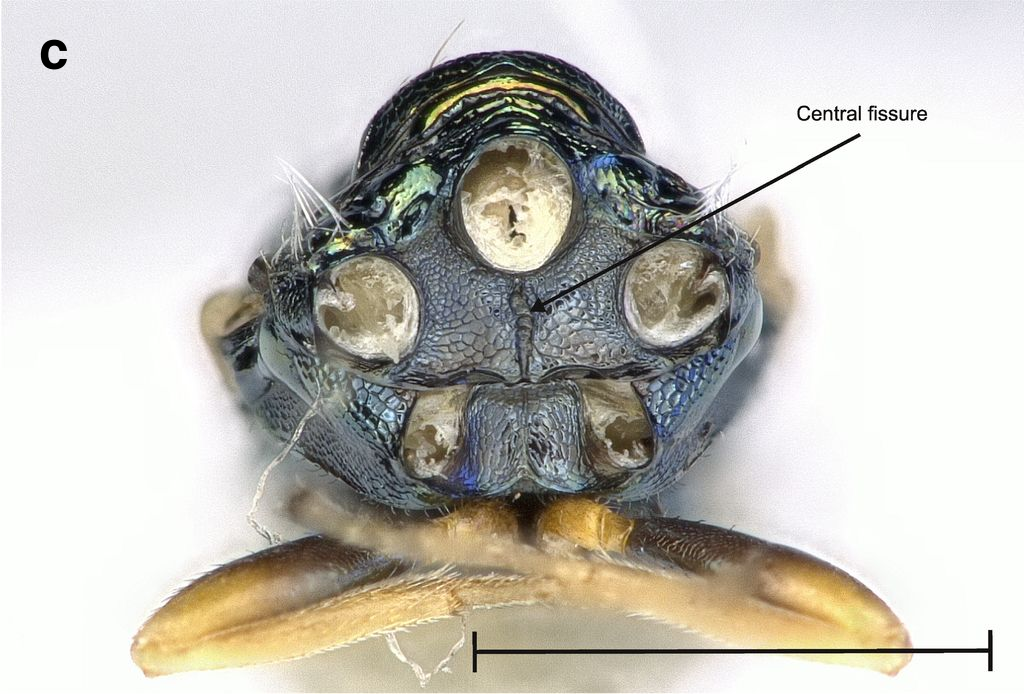
*Pteromalus intermedius*.

**Figure 3a.**
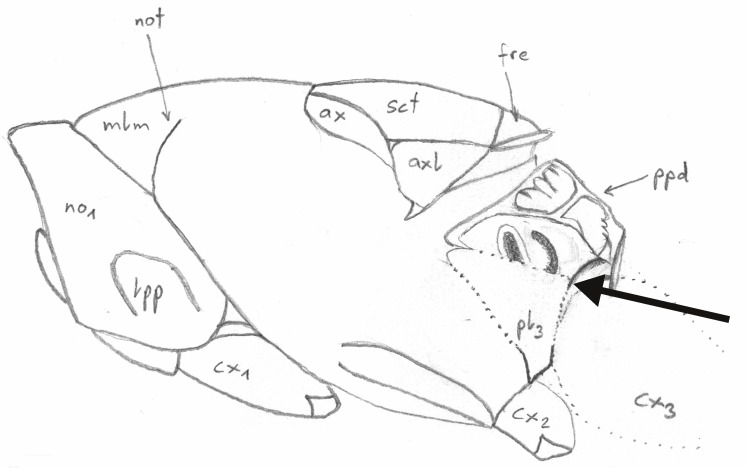
Inclination sharp.

**Figure 3b.**
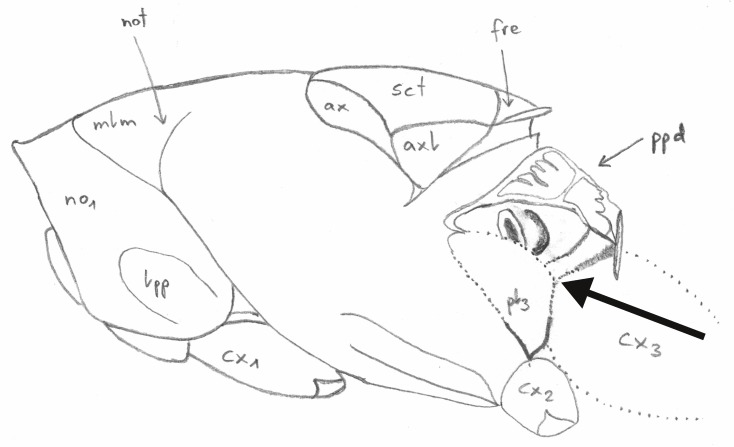
Inclination blunt.

**Figure 4a.**
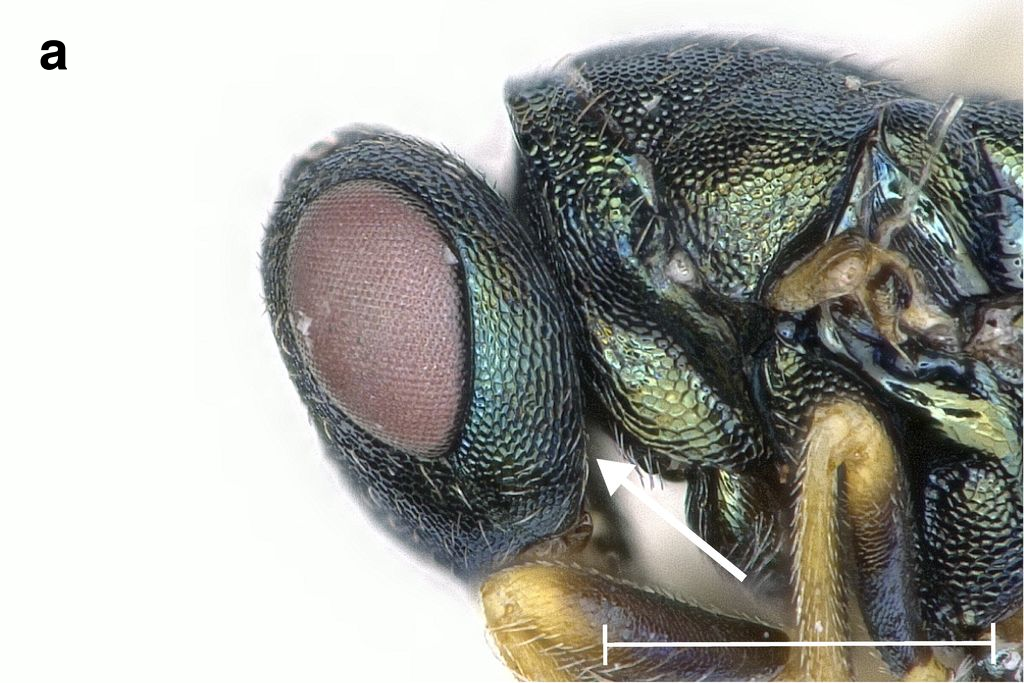
Head, lateral view.

**Figure 4b.**
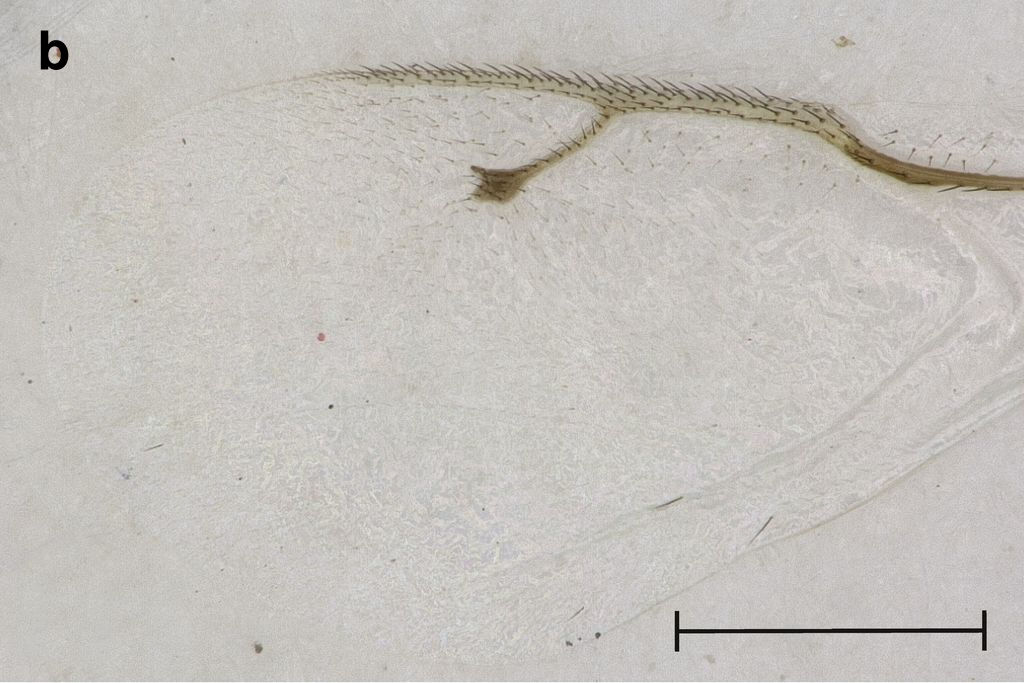
Fore wing, dorsal view.

**Figure 4c.**
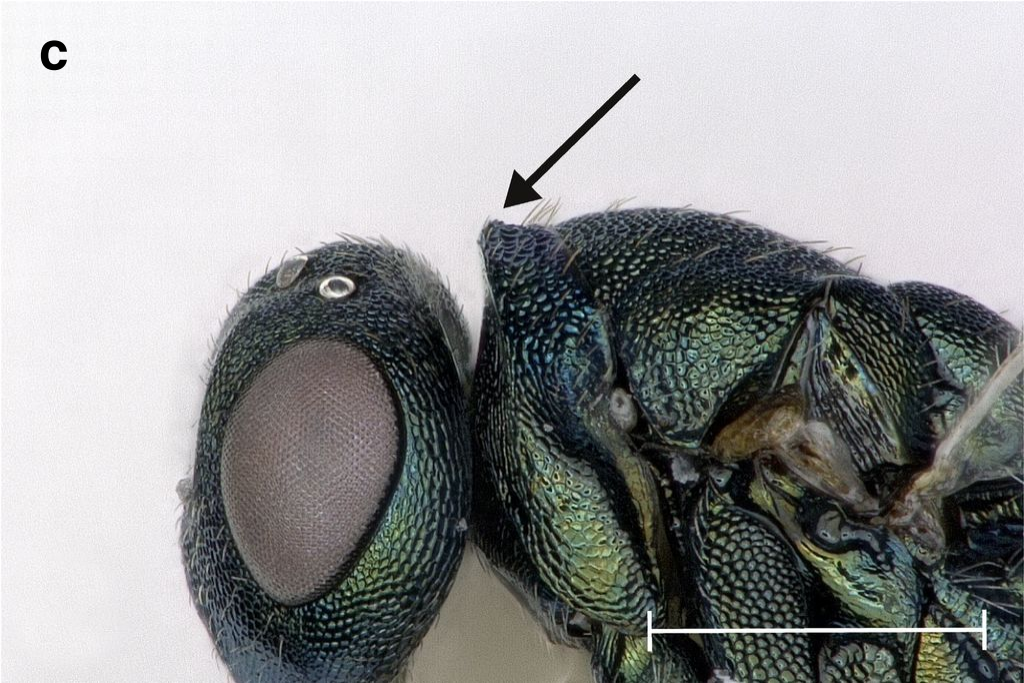
Pronotal collar, lateral view.

**Figure 4d.**
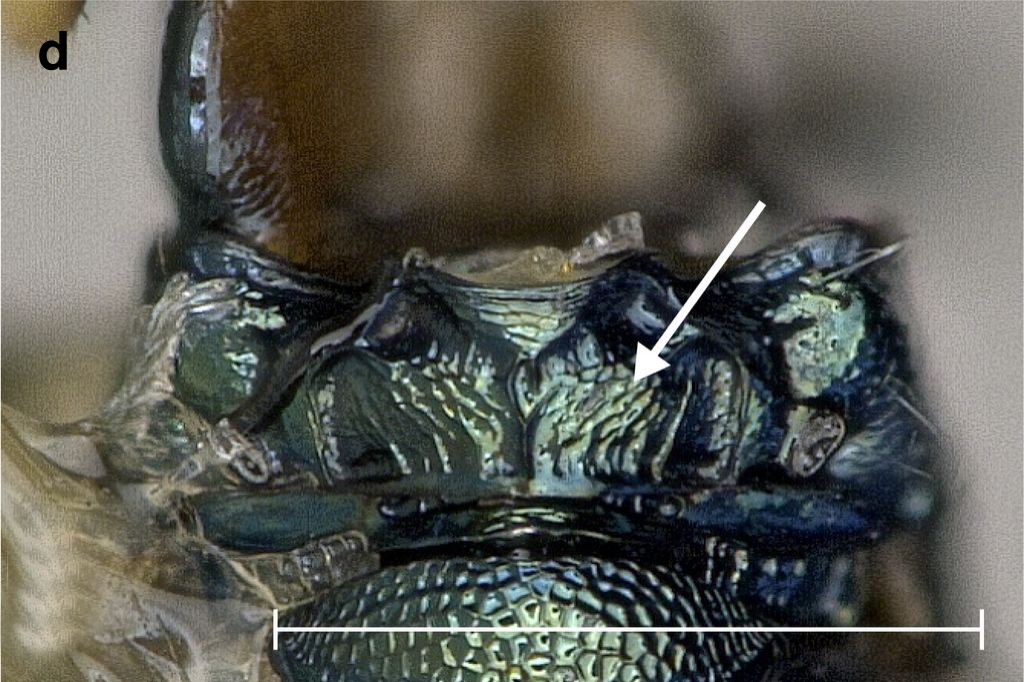
Propodeum, dorsal view.

**Figure 5a.**
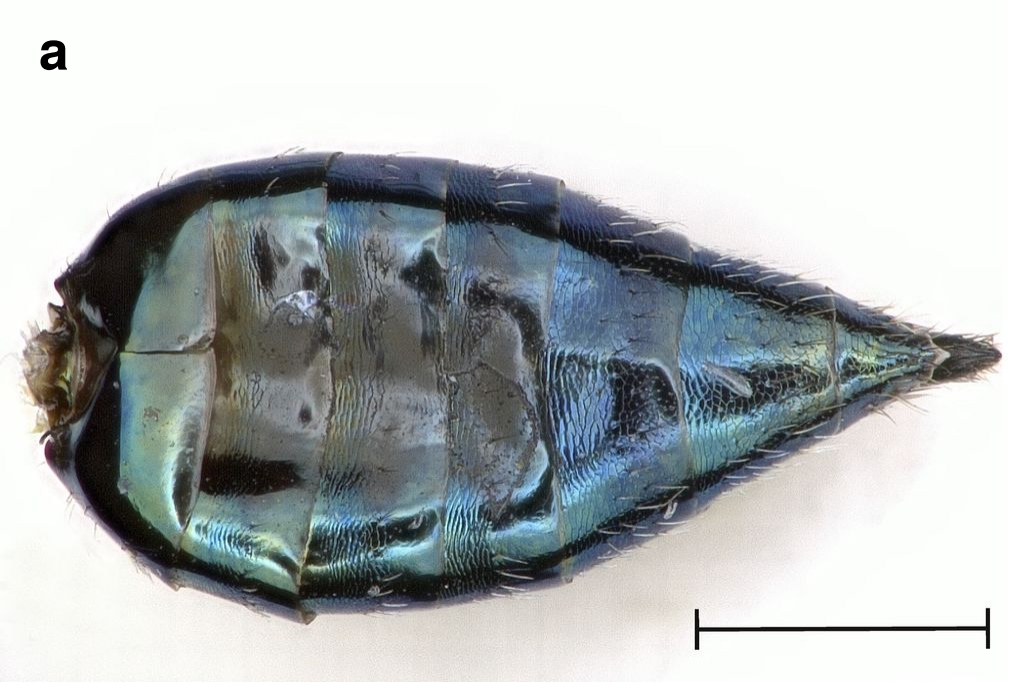
Gaster, dorsal view.

**Figure 5b.**
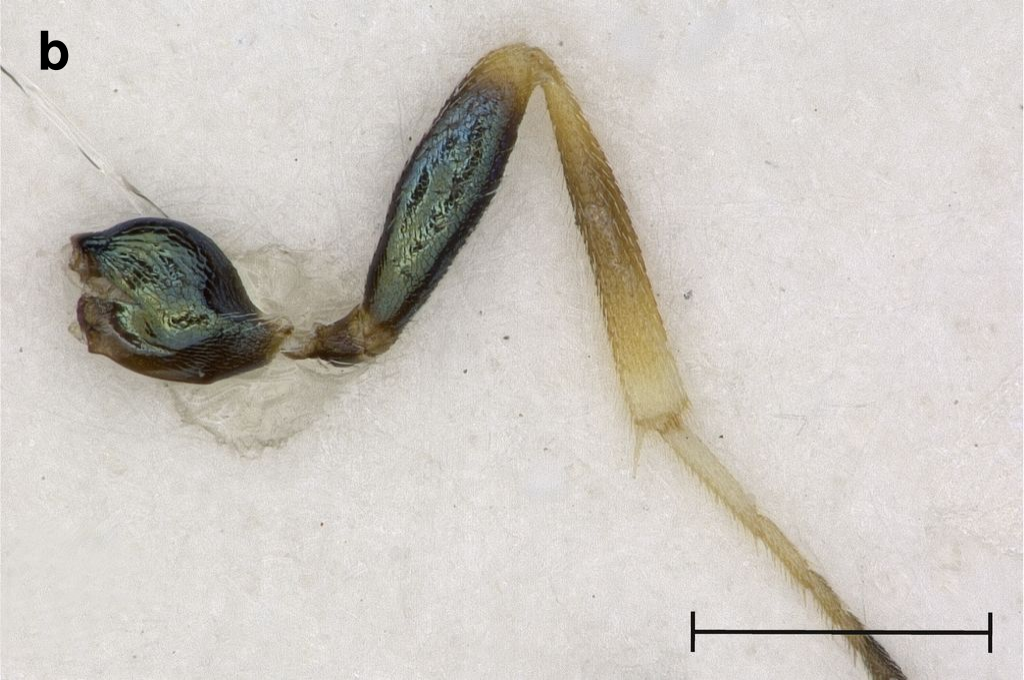
Left metatibia, outer aspect.

**Figure 5c.**
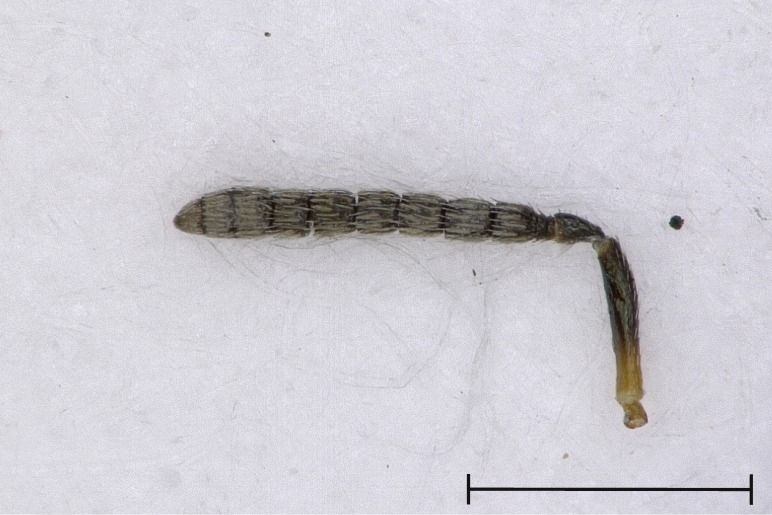
Left antenna, outer aspect.

**Figure 5d.**
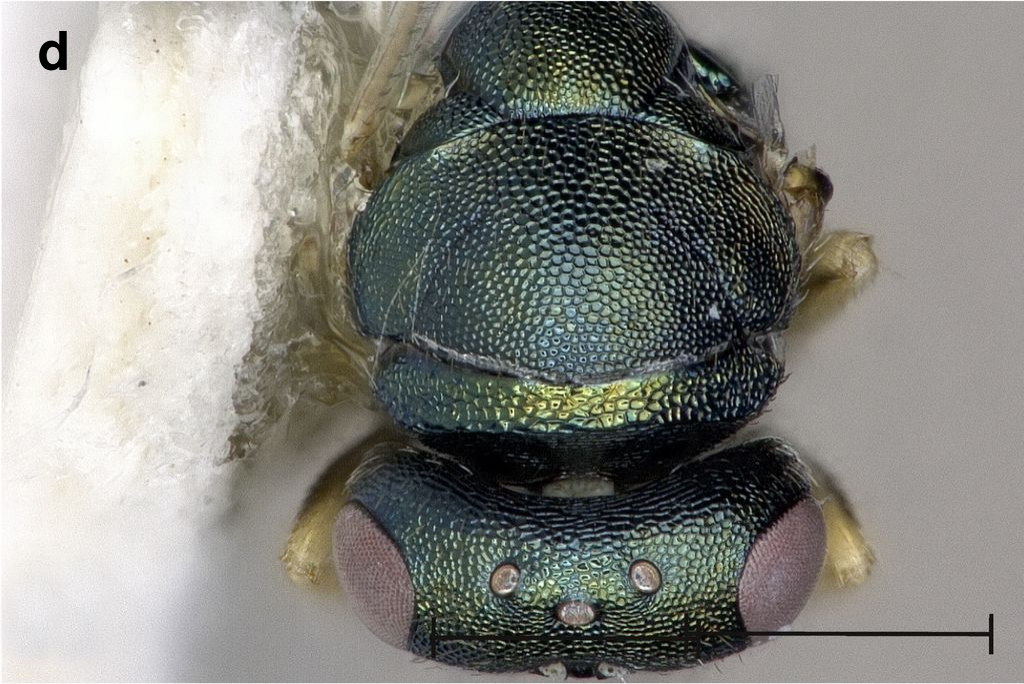
Head and mesoscutum, dorsal view.

**Figure 6a.**
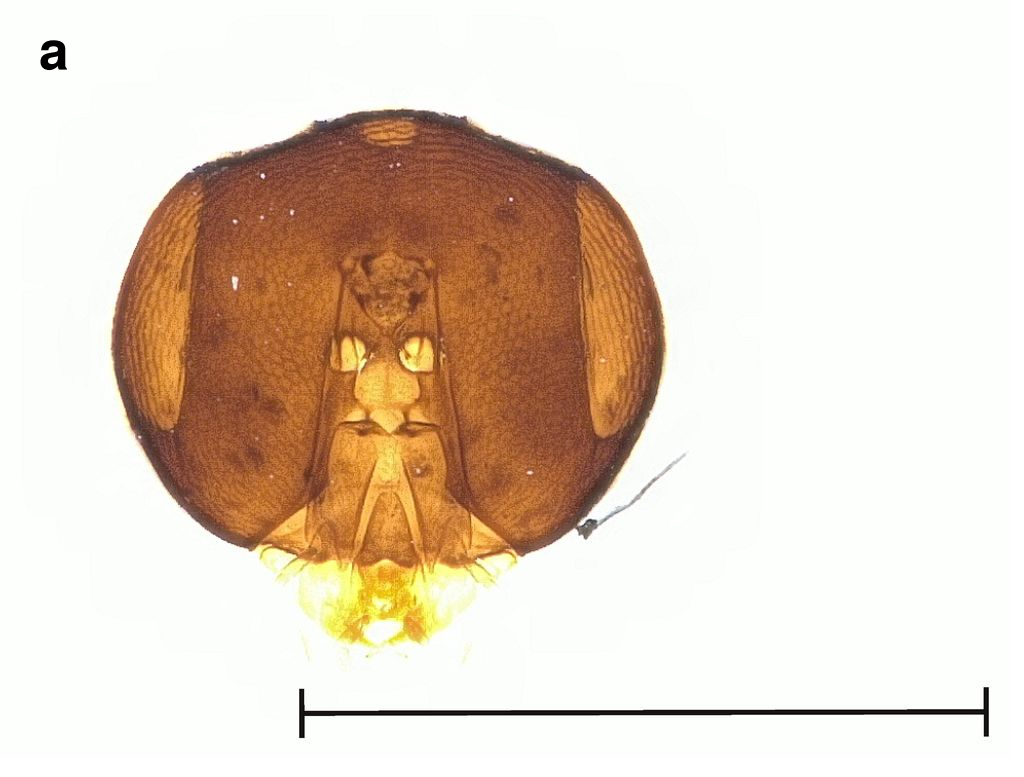
*Pteromalus cingulipes*.

**Figure 6b.**
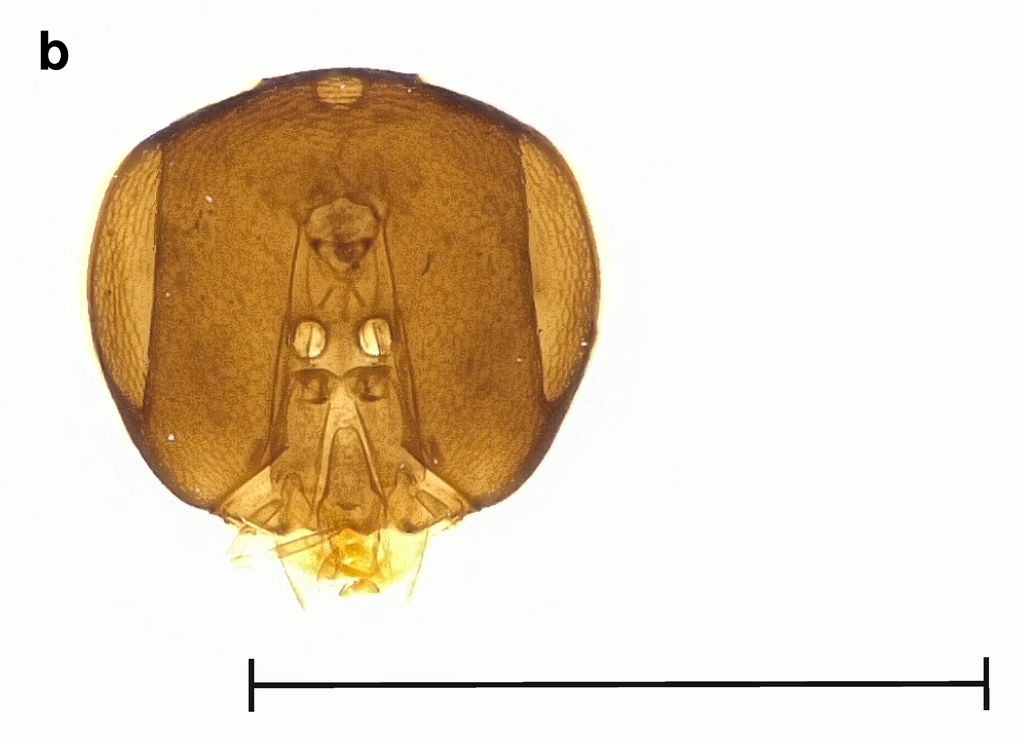
*Pteromalus achillei*.

**Figure 7a.**
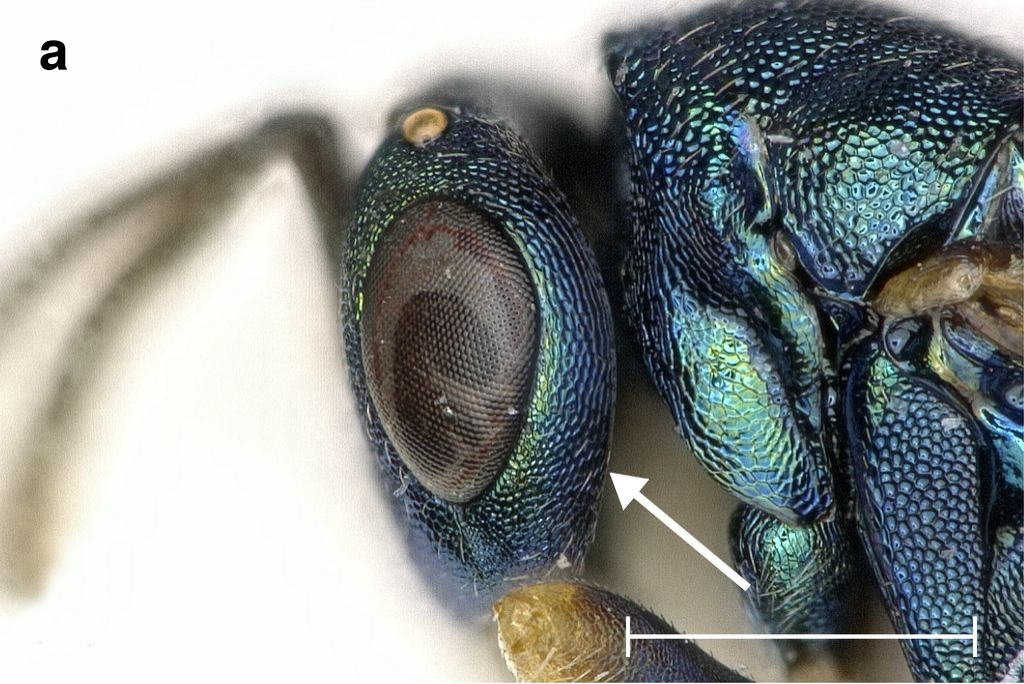
Head, lateral view.

**Figure 7b.**
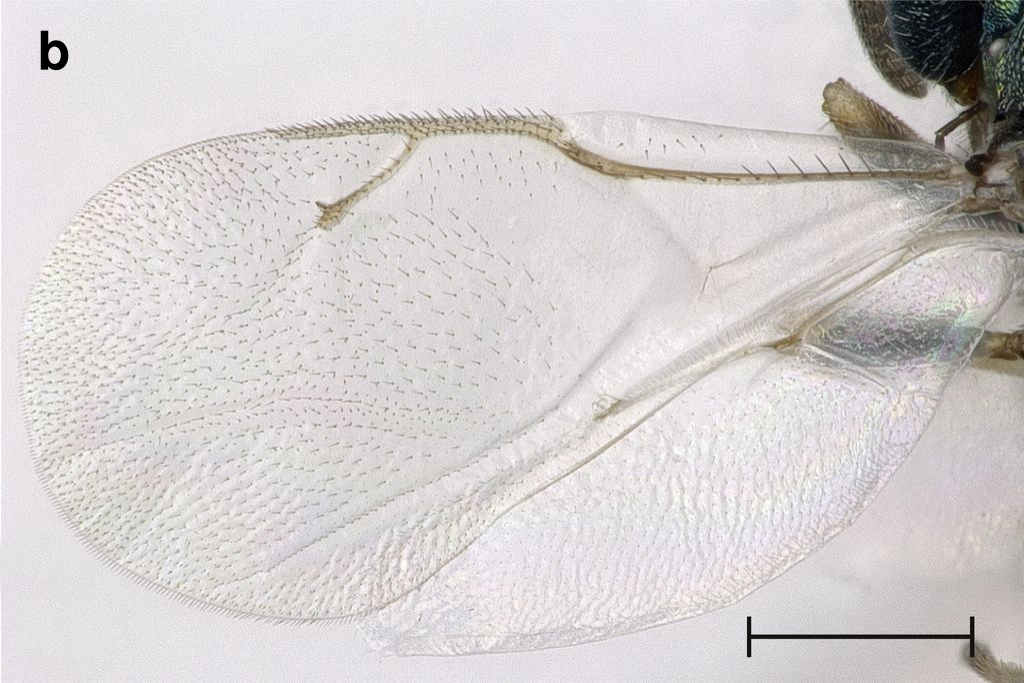
Fore wing, dorsal view.

**Figure 7c.**
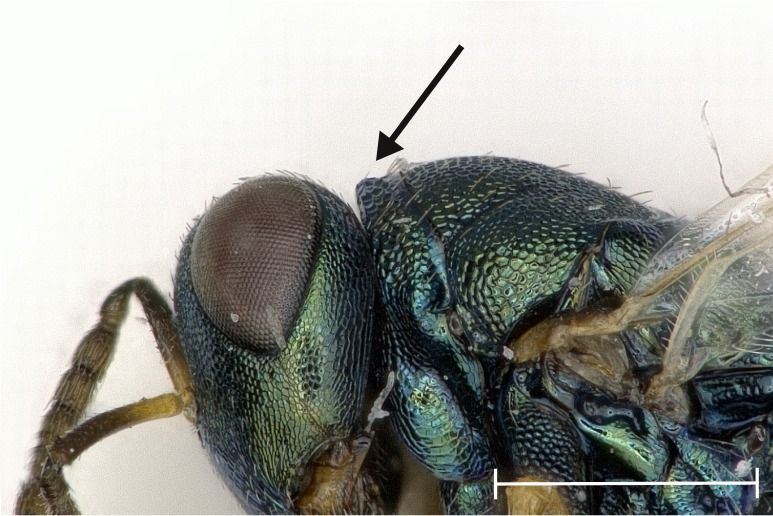
Pronotal collar, lateral view.

**Figure 7d.**
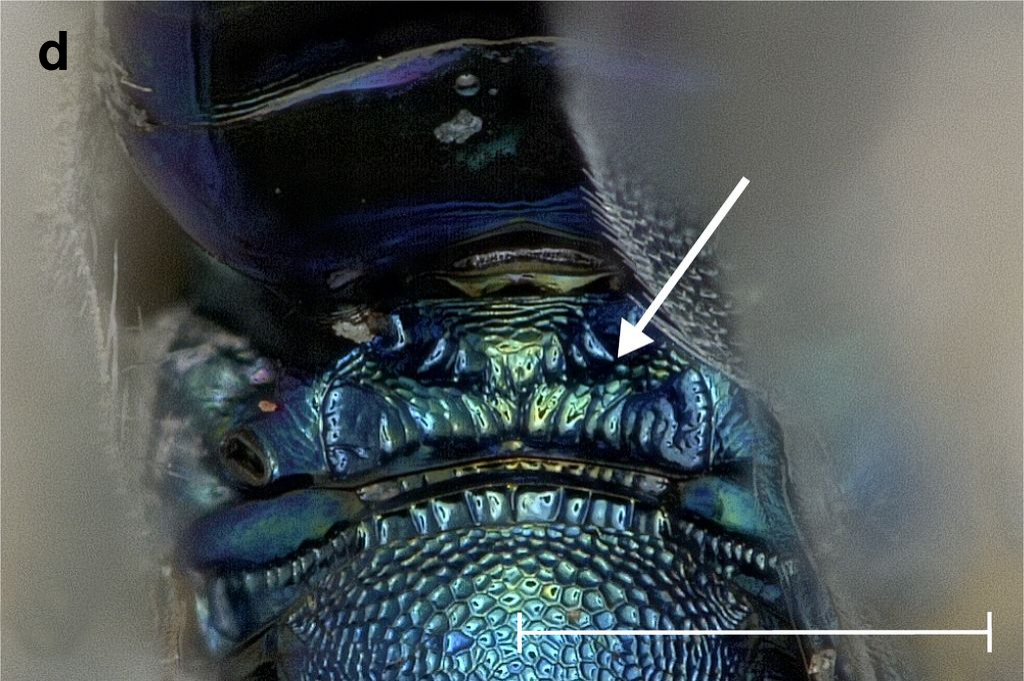
Propodeum, dorsal view.

**Figure 8a.**
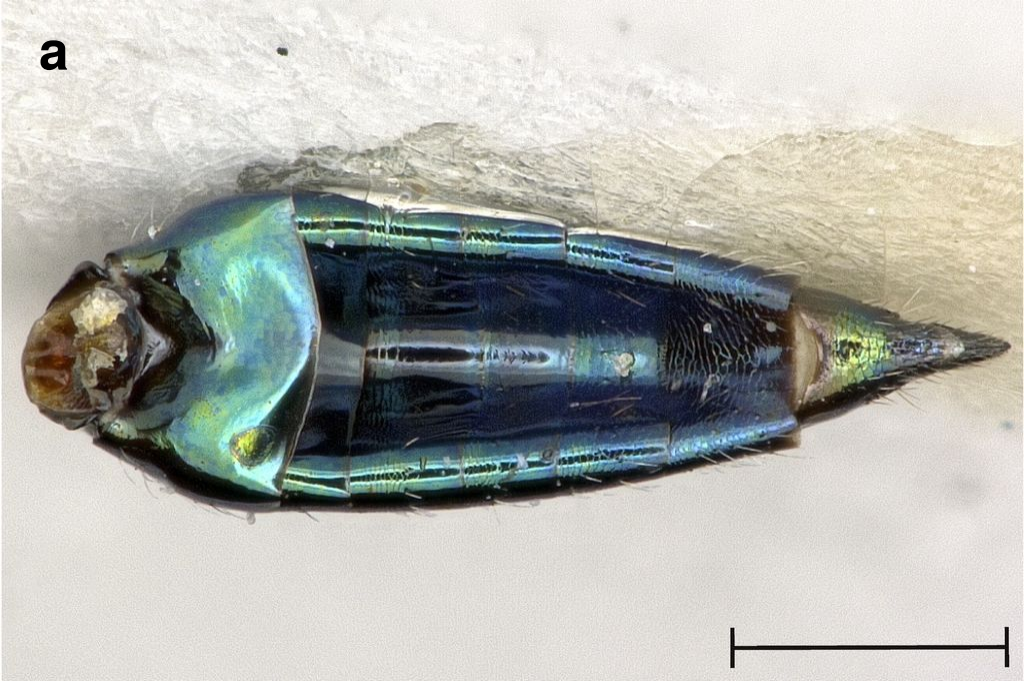
Gaster, dorsal view.

**Figure 8b.**
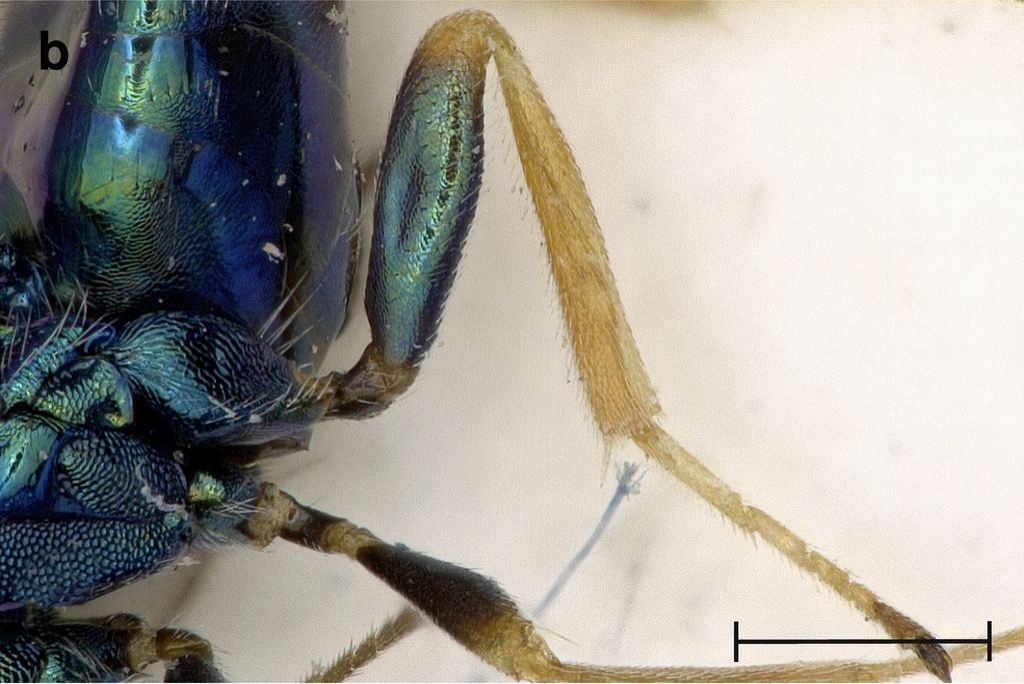
Left metatibia, outer aspect.

**Figure 8c.**
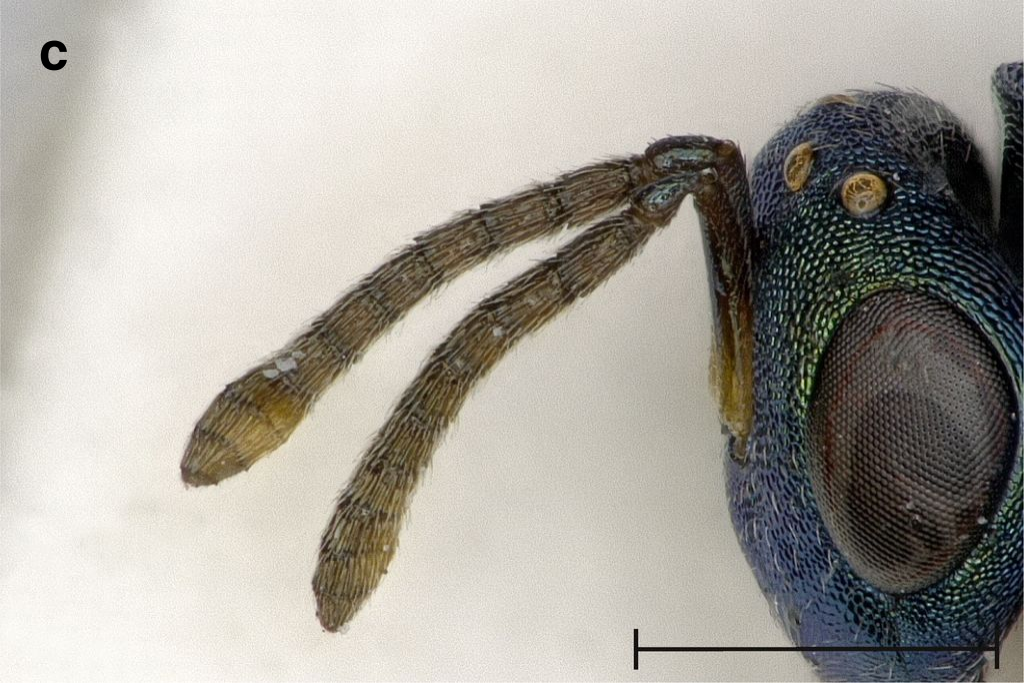
Left antenna, outer aspect.

**Figure 8d.**
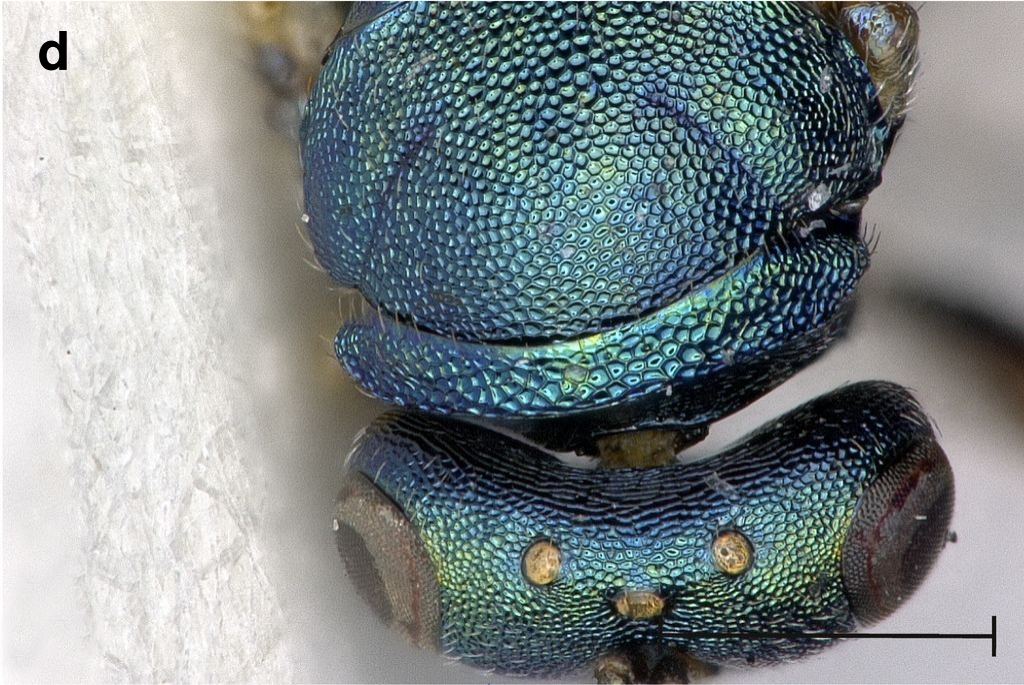
Head and mesoscutum, dorsal view.

**Figure 9a.**
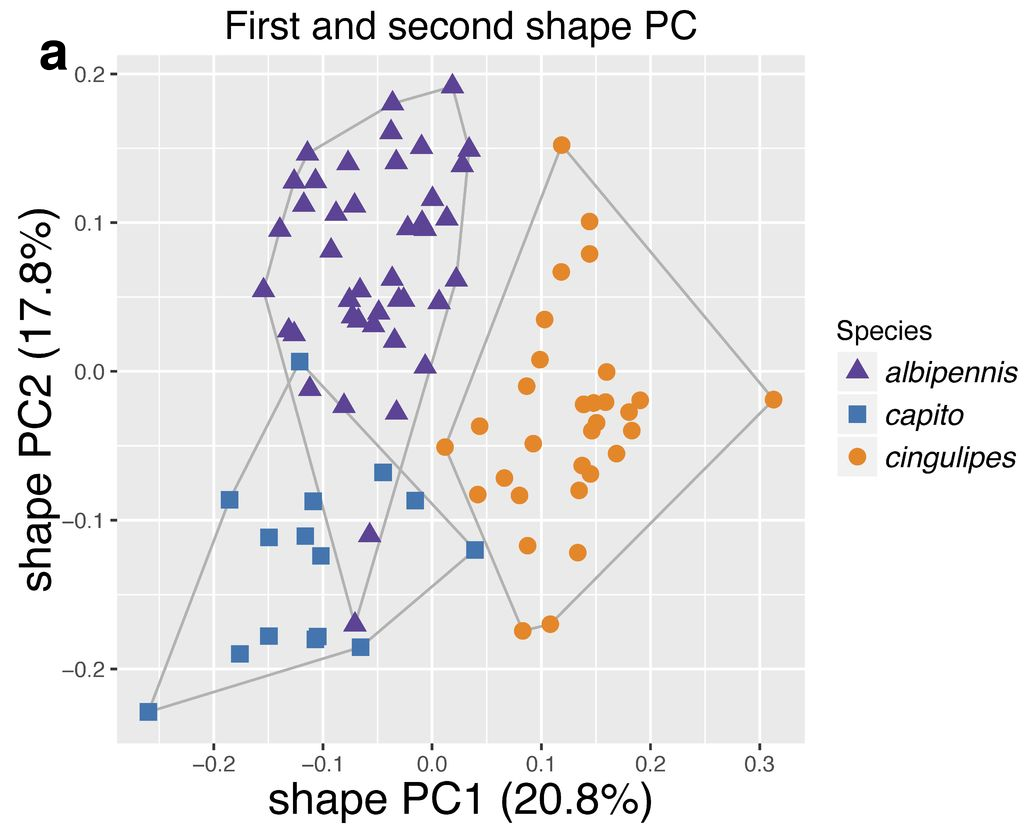
Scatterplot of the first against second shape PC for *Pteromalus albipennis*, *P. capito* and *P. cingulipes*.

**Figure 9b.**
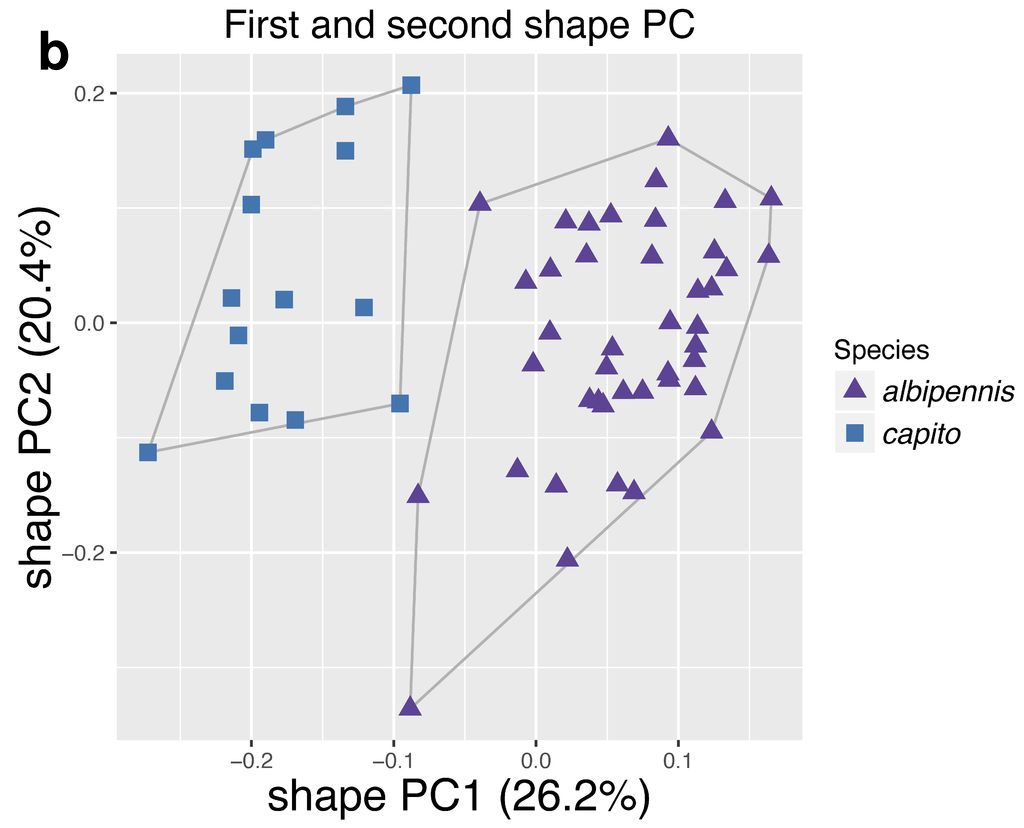
Scatterplot of the first against second shape PC for *Pteromalus albipennis* and *P. capito*.

**Figure 9c.**
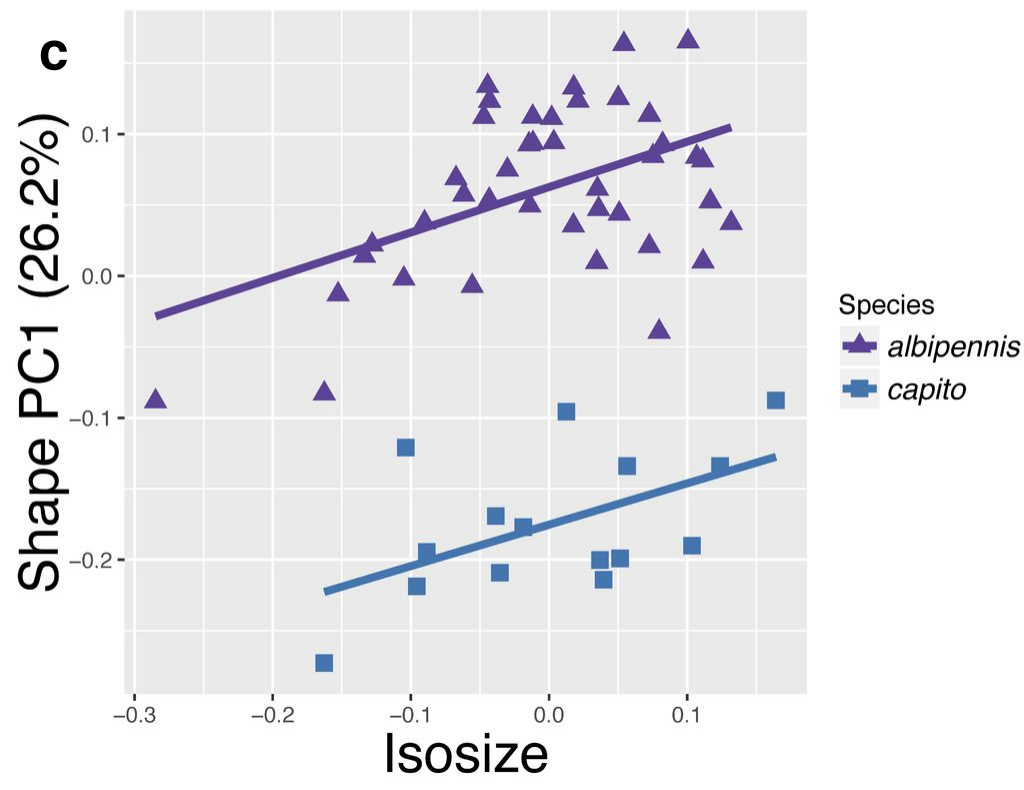
Same species as b, but isosize plotted against first shape PC.

**Figure 9d.**
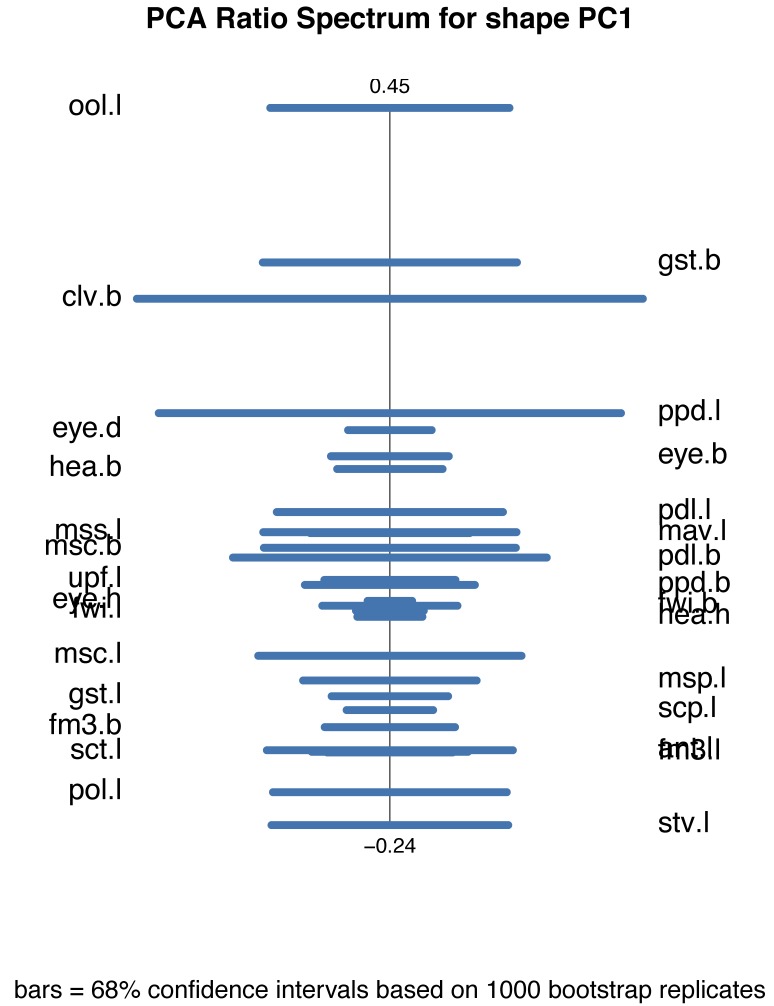
PCA ratio spectrum for the first shape PC as calculated in B and C.

**Table 1.** List of species of the *Pteromalus albipennis* species group and other species included in the key.

**Group**	**Species**
*P. albipennis* species group (29 species)	*aartseni* (Gijswijt, 1972)
	*achillei* Janzon, 1984
	*albipennis* Walker, 1835
	*ametrus* Graham, 1981
	*berylli* Walker, 1835
	*brachygaster* (Graham, 1969)
	*capito* Baur sp.n.
	*cardui* (Erdös, 1953)
	*caudiger* (Graham, 1969)
	*conformis* (Graham, 1969)
	*costulata* Gijswijt, 1999
	*cingulipes* Walker, 1835
	*elevatus* (Walker, 1834)
	*eudecipiens* Özdikmen, 2011 [*P. decipiens* (Graham, 1969)]
	*inclytus* Förster, 1841
	*integer* Walker, 1872
	*intermedius* (Walker, 1834)
	*lactucae* (Szelényi & Erdös, 1953)
	*musaeus* Walker, 1844
	*myopitae* (Graham, 1969)
	*parietinae* (Graham, 1969)
	*patro* Walker, 1848
	*pilosellae* Janzon, 1984
	*scandiae* (Graham, 1969)
	*solidaginis* Graham & Gijswijt, 1991
	*speculifer* Graham, 1981
	*temporalis* (Graham, 1969)
	*tibiellus* Zetterstedt, 1838
	*tripolii* (Graham, 1969)
Other *Pteromalus* (18 species)	*altus* (Walker, 1834)
	*bedeguaris* (Thomson, 1878)
	*bifoveolatus* Förster, 1861
	*briani* Baur, 2015
	*chlorospilus* (Walker, 1834)
	*chrysos* Walker, 1836
	*crassinervis* (Thomson, 1878)
	*cyniphidis* (Linnaeus, 1758)
	*elatus* Förster, 1841
	*hieracii* (Thomson, 1878)
	*janstai* Baur, 2015
	*ochrocerus* (Thomson, 1878)
	*platyphilus* Walker, 1874
	*puparum* (Linnaeus, 1758)
	*semotus* (Walker, 1834)
	*sequester* Walker, 1835
	*vibulenus* (Walker, 1839)
	*vopiscus* Walker, 1839

**Table 2.** Abbreviation, name, definition and magnification of measurements (after Baur 2015). "*Pc*" refers to *Pteromalus capito* Baur sp. n.

**Abbreviation**	**Character name**	**Definition**	**Magnification in pixel/mm**	**Applied to**
ant.l	Antenna length	Combined length of pedicel plus flagellum, outer aspect (Graham 1969)	888	all species
clv.b	Clava breadth	Greatest breadth of clava, outer aspect	888	only *Pc*
eye.b	Eye breadth	Greatest breadth of eye, lateral view	888	only *Pc*
eye.d	Eye distance	Shortest distance between eyes, dorsal view	888	only *Pc*
eye.h	Eye height	Greatest length of eye height, lateral view	888	only *Pc*
eye.l	Eye length	Length of eye, dorsal view (Graham 1969)	888	only *Pc*
fm3.b	Metafemur breadth	Greatest breadth of metafemur, outer aspect	888	all species
fm3.l	Metafemur length	Length of metafemur, from distal end of trochanter to tip of metafemur, measured along midline, outer aspect	888	all species
fwi.b	Fore wing breadth	Greatest breadth of fore wing, measured at about right angle to marginal and postmarginal veins	444	only *Pc*
fwi.l	Fore wing length	Greatest length of fore wing, measured from end of humeral plate to tip of wing	444	only *Pc*
gst.b	Gaster breadth	Greatest breadth of gaster, distance between the outermost lateral edges of the gaster, dorsal view	444	all species
gst.l	Gaster length	Length of gaster along median line from anterior edge of basal tergum to tip of ovipositor sheath, dorsal view	444	all species
hea.b	Head breadth	Greatest breadth of head, dorsal view	888	all species
hea.h	Head height	Distance between anterior margin of clypeus and anterior edge of anterior ocellus, frontal view	888	all species
hea.l	Head length	Length of head, dorsal view (Graham 1969)	888	only *Pc*
mav.l	Marginal vein length	Length of marginal vein, distance between the point at which submarginal vein touches the leading edge of the wing and the point at which the stigma vein and postmarginal vein unite (Graham 1969)	888	all species
msc.b	Mesoscutum breadth	Greatest breadth of mesoscutum just in front of level of tegula, dorsal view	888	all species
msc.l	Mesoscutum length	Length of mesoscutum along median line from posterior edge of pronotum to posterior edge of mesoscutum, dorsal view	888	only *Pc*
msp.l	Malar space	Distance between the point where malar sulcus enters mouth margin and malar sulcus enters lower edge of eye, lateral view (Graham 1969)	888	only *Pc*
mss.l	Mesosoma length	Length of mesosoma along median line from anterior edge of pronotum collar to posterior edge of nucha, dorsal view	888	all species
ool.l	OOL	Shortest distance between posterior ocellus and eye margin, dorsal view (Graham 1969)	888	all species
pdl.b	Pedicel breadth	Greatest breadth of pedicel, outer aspect	888	only *Pc*
pdl.l	Pedicel length	Length of pedicel, outer aspect	888	only *Pc*
plc.d	Plica distance	Greatest distance between upper edge of anterior plica (Baur 2000)	888	all species
pol.l	POL	Shortest distance between posterior ocelli, dorsal view (Graham 1969)	888	all species
ppd.l	Propodeum length	Length of propodeum measured along median line from anterior edge to posterior edge of nucha, dorsal view	888	all species
scp.l	Scape length	Length of scape exclusive of radicle, outer aspect (Graham 1969)	888	only *Pc*
sct.l	Scutellum length	Length of scutellum along median line from posterior edge of mesoscutum to posterior edge of scutellum, dorsal view	888	only *Pc*
stv.l	Stigmal vein length	Length of stigmal vein, distance between the point at which stigma vein and postmarginal vein unite apically and the distal end of the stigma (Graham 1969)	888	all species
tmp.l	Temple length	Length of temple, dorsal view (Graham 1969)	888	only *Pc*
upf.l	Upper face length	Distance between lower edge of toruli and lower edge of anterior ocellus (Gibson et al. 1997)	888	only *Pc*

**Table 3.** Best ratios found by the LDA ratio extractor for delimiting *Pteromalus capito* Baur sp. n. from the most similar species, *P. albipennis* (alb) and *P. cingulipes* (cin).

Species comparison	Best ratio	Value species 1	Value species 2
capito – alb + cin	eye.d : pol.l	>2.73	<2.73
capito – alb	ant.l : ool.l	<5.85	>5.85

## References

[B3769701] Achterberg van Kees (2009). Can Townes type Malaise traps be improved? Some recent developments. Entomologische Berichten, Amsterdam.

[B3769373] Baker M., Shah M., Rosenthal D. S., Roussopoulos M., Maniatis P., Giuli T. J., Bungale P. (2006). A fresh look at the reliability of long-term digital storage.. ACM SIGOPS Operating Systems Review.

[B4115061] Baur H., Austin Andrew, Dowton Mark (2000). Monophyly and relationship of the genus *Coelopisthia* Foerster (Chalcidoidea, Pteromalidae). Hymenoptera: Evolution, Biodiversity and Biological Control.

[B3769641] Baur Hannes, Leuenberger Christoph (2011). Analysis of ratios in multivariate morphometry. Systematic Biology.

[B3769610] Baur Hannes, Kranz-Baltensperger Yvonne, Cruaud Astrid, Rasplus Jean-Yves, Timokhov Alexander V., Gokhman Vladimir E. (2014). Morphometric analysis and taxonomic revision of *Anisopteromalus* Ruschka (Hymenoptera: Chalcidoidea: Pteromalidae) – an integrative approach. Systematic Entomology.

[B3769671] Baur Hannes (2015). Pushing the limits – two new species of *Pteromalus* (Hymenoptera, Chalcidoidea, Pteromalidae) from Central Europe with remarkable morphology. ZooKeys.

[B3769651] Bechshøft T. Ø., Rigét F. F., Wiig Øystein, Sonne Christian (2008). Fluctuating asymmetry in metric traits; a practical example of calculating asymmetry, measurement error, and repeatability. Annales Zoologici Fennici.

[B3769721] Bland J. Martin, Altman Douglas G. (1986). Statistical methods for assessing agreement between two methods of clinical measurement. The lancet.

[B3769914] Bouček Zdenek, Rasplus Jean Yves (1991). Illustrated key to West-Palearctic genera of Pteromalidae (Hymenoptera: Chalcidoidea).

[B4700091] Dallwitz MJ, Paine TA, Zurcher EJ (2018). DELTA – DEscription Language for TAxonomy.

[B3769937] Fisher Nicole, Ubaidillah Rosichon, Reina Placido, La Salle John *Liriomyza* Parasitoids in Southeast Asia. http://keys.lucidcentral.org/keys/v3/Liriomyza/.

[B3769979] Gebiola M, Monti M. M., Johnson R. C., Woolley J. B., Hunter M. S., Giorgini M., Pedata P. A. (2017). A revision of the *Encarsia
pergandiella* species complex (Hymenoptera: Aphelinidae) shows cryptic diversity in parasitoids of whitefly pests. Systematic Entomology.

[B3769711] Gibson Gary A. P., Huber John T., Woolley James B. (1997). Annotated Keys to the Genera of Nearctic Chalcidoidea (Hymenoptera). NRC Research Press.

[B3769771] Gibson Gary A. P. (2015). The presence of *Notanisus* Walker (Hymenoptera: Pteromalidae) in North America and revision of the *oulmesiensis* species group. Zootaxa.

[B3770003] Gijswijt M. J. (1972). Dutch Chalcids VIII. Entomologische. Berichten.

[B3769751] Gijswijt M. J. (1999). Four new species of *Pteromalus* Swederus (Hymenoptera: Chalcidoidea: Pteromalidae) and redescriptions of three other species. Zoologische Mededeelingen.

[B3769731] Graham M W R de V (1969). The Pteromalidae of North-Western Europe. Bulletin of the British Museum (Natural History), Entomology, Supplement.

[B3770023] Graham M. W. R. d. V., Gijswijt M. J. (1991). A new species of *Pteromalus* (Hymenoptera: Chalcidoidea) from France, associated with *Solidago
virgaurea*. Entomologische Berichten.

[B3769791] Grissell Eric (1995). Toryminae (Hymenoptera: Chalcidoidea: Torymidae).

[B3770058] Janzon L. Å. (1984). Taxonomy of a new subgroup of the *Pteromalus
albipennis* group and their host relationships, with description of four new species (Hymenoptera: Chalcidoidea). Paper VI. Taxonomical and biological studies of *Tephritis* species (Diptera) and their parasitoids (Hymenoptera).

[B3770091] Nature Key to http://www.keytonature.eu/wiki/XPER_depository.

[B3769781] Krogmann Lars, Burks Roger A. (2009). *Doddifoenus
wallacei*, a new giant parasitoid wasp of the subfamily Leptofoeninae (Chalcidoidea: Pteromalidae), with a description of its mesosomal skeletal anatomy and a molecular characterization. Zootaxa.

[B3769661] László Zoltán, Baur Hannes, Tóthmérész Béla (2013). Multivariate ratio analysis reveals *Trigonoderus
pedicellaris* Thomson (Hymenoptera, Chalcidoidea, Pteromalidae) as a valid species. Systematic Entomology.

[B3769691] Lotfalizadeh Hosseinali, Delvare Gerard, Rasplus J. Y. (2007). Phylogenetic analysis of Eurytominae (Chalcidoidea: Eurytomidae) based on morphological characters. Zoological Journal of the Linnean Society.

[B3770110] Lucidcentral.org http://keyserver.lucidcentral.org/key-server/keys.jsp.

[B3769761] Lutz Lena (2014). Taxonomic differentiation of three species of the *Pteromalus
albipennis* species group (Hymenoptera: Chalcidoidea: Pteromalidae), using an integrative approach. Bachelor thesis, Rheinische-Fridrich-Wilhelms Universität, Bonn.

[B3770129] Maletti Sina, Peters Ralph, Schmidt Stefan, Traunmüller Franziska, Baur Hannes (in press). From morphometry to taxonomy – using multivariate analyses for the evaluation and characterization of cryptic species.. Insect Systematics & Evolution.

[B3770160] Marsh Paul. M., Wild Alexander. L. An interactive key to the *Heterospilus* (Hymenoptera: Braconidae: Doryctinae) of Costa Rica. http://keyserver.lucidcentral.org/key-server/player.jsp?keyId=106.

[B3769681] Noyes John S. (1982). Collecting and preserving chalcid wasps (Hymenoptera: Chalcidoidea). Journal of Natural History.

[B3770178] Noyes John S. Universal Chalcidoidea Database. World Wide Web electronic publication. http://www.nhm.ac.uk/chalcidoids.

[B3770197] Team R Core (2013). R: A language and environment for statistical computing. http://www.R-project.org.

[B3771794] Schneider C. A., Rasband W. S., Eliceiri K. W. (2012). NIH Image to ImageJ: 25 years of image anaylsis. Nature Methods.

[B4700101] Vignes-Lebbe Régine, Bouquin Sylvain, Kerner Adeline, Bourdon Estelle (2017). Desktop or remote knowledge base management systems for taxonomic data and identification keys: Xper2 and Xper3. Proceedings of TDWG.

[B3770216] Wickham H. (2016). ggplot2: Elegant Graphics for Data Analysis.

[B3769741] Wynford-Thomas David, Jasani Bharat, Newman Geoffrey R. (1986). Immunohistochemical localization of cell surface receptors using a novel method permitting simple, rapid and reliable LM/EM correlation. The Histochemical Journal.

